# CXCR2 expression during melanoma tumorigenesis controls transcriptional programs that facilitate tumor growth

**DOI:** 10.1186/s12943-023-01789-9

**Published:** 2023-06-03

**Authors:** J. Yang, K. Bergdorf, C. Yan, W. Luo, S. C. Chen, G.D. Ayers, Q. Liu, X. Liu, M. Boothby, V.L. Weiss, S. M. Groves, A. N. Oleskie, X. Zhang, D. Y. Maeda, J. A. Zebala, V. Quaranta, A. Richmond

**Affiliations:** 1 TVHS Department of Veterans Affairs, Nashville, TN 37212 USA; 2 Present Address: Department of Pharmacology, Vanderbilt University School of Medicine, Nashville, TN 37240 USA; 3 Department of Biostatistics, Vanderbilt University Medical Center, Nashville, TN 37203-1742 USA; 4 Department of Pathology, Microbiology, and Immunology, Vanderbilt University Medical Center, Nashville, TN 37232 USA; 5 Department of Genomic Medicine, MD Anderson Cancer Center, University of Texas, Houston, TX 77030 USA; 6 Syntrix Pharmaceuticals, Auburn, WA 98001 USA; 7 Department of Biochemistry, Vanderbilt University, TN 37240 Nashville, USA

**Keywords:** Melanoma, CXCR2, Tumor immune microenvironment, Genomic analysis, Genetic mouse models

## Abstract

**Background:**

Though the CXCR2 chemokine receptor is known to play a key role in cancer growth and response to therapy, a direct link between expression of CXCR2 in tumor progenitor cells during induction of tumorigenesis has not been established.

**Methods:**

To characterize the role of CXCR2 during melanoma tumorigenesis, we generated tamoxifen-inducible tyrosinase-promoter driven *Braf*^*V600E*^*/Pten*^*−/−*^*/Cxcr2*^*−/−*^ and *NRas*^*Q61R*^*/INK4a*^*−/−*^*/Cxcr2*^*−/−*^ melanoma models. In addition, the effects of a CXCR1/CXCR2 antagonist, SX-682, on melanoma tumorigenesis were evaluated in *Braf*^*V600E*^*/Pten*^*−/−*^ and *NRas*^*Q61R*^*/INK4a*^*−/−*^ mice and in melanoma cell lines. Potential mechanisms by which *Cxcr2* affects melanoma tumorigenesis in these murine models were explored using RNAseq, mMCP-counter, ChIPseq, and qRT-PCR; flow cytometry, and reverse phosphoprotein analysis (RPPA).

**Results:**

Genetic loss of *Cxcr2* or pharmacological inhibition of CXCR1/CXCR2 during melanoma tumor induction resulted in key changes in gene expression that reduced tumor incidence/growth and increased anti-tumor immunity. Interestingly, after *Cxcr2* ablation, *Tfcp2l1*, a key tumor suppressive transcription factor, was the only gene significantly induced with a log_2_ fold-change greater than 2 in these three different melanoma models*.*

**Conclusions:**

Here, we provide novel mechanistic insight revealing how loss of *Cxcr2* expression/activity in melanoma tumor progenitor cells results in reduced tumor burden and creation of an anti-tumor immune microenvironment. This mechanism entails an increase in expression of the tumor suppressive transcription factor, *Tfcp2l1,* along with alteration in the expression of genes involved in growth regulation, tumor suppression, stemness, differentiation, and immune modulation. These gene expression changes are coincident with reduction in the activation of key growth regulatory pathways, including AKT and mTOR.

**Supplementary Information:**

The online version contains supplementary material available at 10.1186/s12943-023-01789-9.

## Introduction

Chemokines and their receptors have been shown to play an essential role in regulating tumor growth, progression, metastasis, and response to immunotherapy [1–4]. Though chemokines were initially identified as chemoattractants used to guide leukocyte migration, there has been increasing evidence that they can regulate other functions in a broader array of cell types, including cancer cells [5].

The CXCR1/CXCR2 ligand-receptor axis has been widely characterized as a driver of aggressive behavior in many cancer types, including breast, prostate, melanoma, lung, colorectal, pancreatic, and renal cancers [6]. CXCR1/CXCR2 ligands, including CXCL1-3, 5–8 are produced by endothelial cells, tumor-associated macrophages, cancer-associated fibroblasts, adipocytes, and cancer cells [6]. These CXCR1 and CXCR2 ligands play a significant role in the recruitment of neutrophils and myeloid-derived suppressor cells (MDSCs) to the tumor microenvironment (TME), both of which are associated with poor outcomes [7–9]. In addition to altering the tumor immune microenvironment, these chemokine ligands can also activate phosphatidylinositol-3-kinase (PI3K), phospholipase-Cβ, calcium mobilization, mitogen-activated protein kinase (MAPK), protein kinase B (AKT), transcription factors like NF-κB, and gene expression on tumor cells. These chemokine responses have been linked to tumor cell survival, proliferation, migration, as well as angiogenesis [6, 10, 11].

Many cancer cells exhibit induction or increased expression of multiple ligands for both CXCR1 (CXCL1-3, 5–8) and CXCR2 (CXCL1-3, 5 and 7). Moreover, CXCR1 and CXCR2 are differentially expressed in human tissues, though in mouse, CXCR2 is the predominant receptor mediating response to the murine chemokine ligands during inflammation, angiogenesis, and tumor growth (CXCL1,2,3 and 5, also known as KC, MIP2α, MIP2β, and LIX) [12, 13]. In addition to a function in the attraction of hematopoietic cells that influence the tumor microenvironment and tumor progression, it has been suggested that these receptors may exert autocrine effects on tumor growth. In the case of melanoma, mouse xenograft models provide compelling evidence that tumor cells take advantage of CXCR2 ligand expression to either suppress the anti-tumor immune response or to induce tumor growth and angiogenesis, alter the TME, and facilitate metastasis [3, 14].

The CXCR1/CXCR2 signaling nexus directly influences the sensitivity of tumor cells to chemotherapies by altering pathways associated with apoptosis and multidrug resistance [15, 16], resulting in a poor prognosis in human cancer studies [17, 18]. The past decade has witnessed the generation and development of antagonists to CXCR1 and CXCR2, and multiple clinical trials are underway investigating the therapeutic potential of targeting this signaling axis in inflammatory disorders and cancers (NCT03161431, NCT04245397, NCT03400332) [19–22].

We previously demonstrated that targeted deletion of *Cxcr2* in myeloid cells or systemic treatment with the CXCR1/CXCR2 antagonist SX-682 conferred anti-tumor immunity via reduction of MDSC infiltration into the TME and enhanced CD8 + T cell activation [9]. However, it remains controversial as to whether there is a direct function of either or both CXCR1 and CXCR2 on the growth of the cancer cells, and if so, which of these receptors are involved and what mechanisms are employed. To clarify the concept of an autocrine role for CXCR2 and its ligands in melanoma progenitor cells, we used inducible, autochthonous models of malignant melanoma in mice. Using two distinct modes of triggering the formation of malignant melanoma [23, 24], we found that tumor onset, growth, and outcome accompanied changes in the tumor microenvironment (TME) and gene expression when *Cxcr2* was deleted in melanoma precursor cells. Similar results were identified when Cxcr1/Cxcr2 were inhibited with SX-682 during tumorigenesis. Remarkably, an analysis of common gene expression changes due to loss or inhibition of Cxcr2 during tumorigenesis converged on one, but only one, gene – the tumor suppressive transcription factor *Tfcp2l1*. These data indicate that one mechanism by which Cxcr2 inhibition regulates melanoma tumor growth is via induction of a key transcription factor, *Tfcp2l1. Tfcp2l1 is* a member of the Tfcp2/Tfcp2l1/Ubp1 subfamily of Grainyhead-like transcription factors. These transcription factors all bind to the same DNA sequences and regulate numerous functions including differentiation, tumor suppression, regeneration, stemness, drug metabolism, regulation of blood pressure and water channel function [25–28].

## Methods

### Establishment of inducible melanoma mouse models

All procedures involving animals were approved by the Vanderbilt University Institutional Animal Care and Use Committee (IACUC). We utilized the inducible *Braf*^*V600E*^*/PTEN*^*−/−*^ melanoma model in C57BL/6 mice [23], where the underlying genetic background includes *Tyr-Cre*^*ER*+^*:: Braf*^*CA*^*::Pten*^*lox4*−5/Lox4−5^. CXCR2^f/f^ mice *(C57BL/6-CXCR2*^*tm1RMra/J*^*)* were obtained from Jackson Laboratories (#024638) and bred to mT/mG mice (#007907, Jackson Laboratories), which harbor a two-color fluorescent Cre-reporter allele to enable GFP-based tumor imaging (Figure S2A,C) [29]. In crossing the *Braf*^*V600E*^*/PTEN*^*−/−*^ mice with CXCR2^fl/fl^ mT/mG mice, Tyr-*Cre*^*ER*+^*::Braf*^*CA*^*::Pten*^*lox4−5/Lox4−5*^*::mT/mG::Cxcr2*^*fl/fl*^ mice and *Cre*^*ER*+^*:: Braf*^*CA*^*::Pten*^*lox4−5/Lox4−5*^*::mT/mG::Cxcr2*^*WT*^ mice were generated*.* Upon administration of 4-HT (#6278, Sigma), Cre-recombinase expression is induced in tyrosinase (*Tyr*) expressing cells, leading to expression of the *Braf*^*V600E*^ transgene and deletion of exons 4 and 5 of *Pten* specifically in tyrosinase expressing melanocytes (Figure S2B) [23]. Palpable tumors arise within one month post 4-HT induction (Figure S2B, C). Tyr-Cre targeting of melanocytes in hair follicles was verified by H&E staining and GFP expression (Figure S2D).

To generate an inducible *NRas* mutant/*Ink4a* deletion/CXCR2 knockout melanoma mouse model, we utilized the *TpN*^*61R*^ model from Burd et al., which recapitulates the genetics of *NRAS*^*Q61R*^*/INK4a*^*−/−*^ mutant human melanoma and demonstrates sensitivity to UV-induced melanoma [30]. In this model, expression of mutant *NRas* and loss of *Ink4a* are under the control of the *Tyr*-promoter enhancer *(Tyr-Cre*^*ER*^*::NRas*^*Q61R*^*::Ink4a*^*−/−*^*).* These mice were crossed with C57BL/6 *Cxcr2*^*f/f*^ mice. Heterozygous offspring were crossed to generate *Tyr-Cre*^*ER*^*::NRas*^*Q61R*^*::Ink4a*^*fl/fl*^*::Cxcr*^*fl/fl*^* and Tyr-Cre*^*ER*^*::NRas*^*Q61R*^*::Ink4a*^*fl/fl*^*::Cxcr2*^*WT*^ littermates*.* Newborn mice (1–2 days of age) receive one topical administration of 2 μl of 20 mM 4HT on the back followed by exposure to 4.5 K J/m^2^ UVB radiation (312NM 2X8 Watt tubes& Filter, Cat. # EB-280C) on day three. Tumor development was followed for 5 months. All other standard methods are in the Supplemental Materials.

## Results

### CXCR2 correlates with poor prognosis in patient populations and response to checkpoint inhibitors

**Fig. 1:**
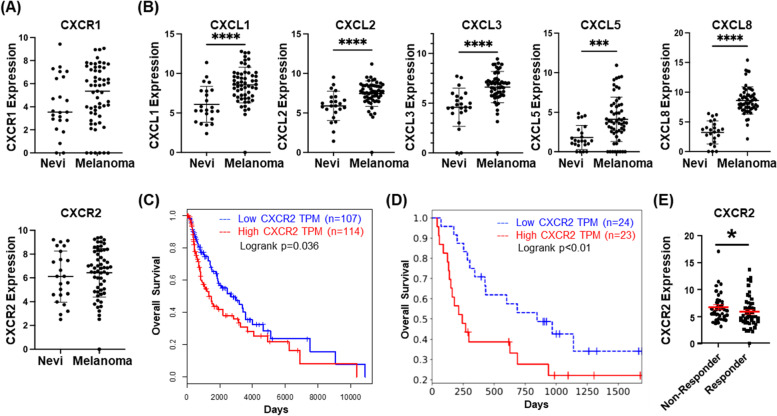
CXCR2 is associated with tumorigenesis and poor prognosis. **a** GEO dataset analysis of expression of CXCR1 and CXCR2 in nevi as compared to melanoma lesions (not significant, Welch's t-test). **b** GEO dataset analysis of expression of CXCL1, CXCL2, CXCL3, CXCL5 and CXCL8 in nevi and melanoma tissues (significance determined by Welch's t-test). **c** Overall survival plot of melanoma patients from the TCGA SKCM dataset indicates significantly improved survival (*p *= 0.035, log-rank test) in the lowest quartile of CXCR2 expression (blue, *n* = 107) compared to the highest quartile (red, *n* = 114). **d** Analysis of survival of 25 melanoma patients treated with anti-PD-1 in relation to high (red) or low (blue) expression of CXCR2 [*p* < 0.01, log-rank test; [27]]. **e** Re-analysis of the Riaz RNA-seq database shows CXCR2 expression is lower in melanoma patients who responded to anti-PD1 treatment (*p* < 0.05, Welch's t-test)

Using the available Gene Expression Omnibus (GEO) cohort, we evaluated *CXCR1, CXCR2,* and *CXCL1-3, 5* and* 8* (CXCR1/CXCR2 ligands) expression in nevi and melanoma. *CXCR1* and *CXCR2* mRNA exhibited a trend toward increased expression in melanoma compared to nevi, but these differences were not statistically significant (Fig. 1A). This may be partially explained by the analysis being performed on bulk RNA-sequencing data, rather than measuring tumor cell-specific expression. However, C*XCL1*, *CXCL2*, *CXCL3*, *CXCL5* and *CXCL8* mRNAs were significantly upregulated in melanoma samples compared to benign nevi (Fig. 1B). Furthermore, there were no significant differences in *CXCR1* and *CXCR2* expression among nevi and melanoma tumors when stratified by *BRAF* or *NRAS* mutation status (Figure S1A, B). However, since the number of samples available for analysis of mutation status was small, these findings should be interpreted cautiously.

CXCR2 has been associated with increased tumor growth and poor prognosis across multiple cancers [6]. To define the relationship between *CXCR2* expression and the clinical prognosis of melanoma patients, we examined clinical data from the Cancer Genome Atlas (TCGA), and the skin cutaneous melanoma (SKCM) dataset using Gene Expression Profiling Interactive Analysis (GEPIA). Survival analysis comparing patients with high *CXCR2* expression (*n* = 114) to patients with lower *CXCR2* expression (*n* = 107) indicates that *CXCR2* expression correlates with decreased overall survival of melanoma patients (*p* = 0.035, Fig. 1C). Evaluation of survival in a patient cohort treated with anti-PD-1 therapy also suggests that patients with high *CXCR2* expression (*n* = 24) exhibited poor prognosis in response to anti-PD-1 when compared with patients with low *CXCR2* expression (*n* = 23, *p* < 0.01; Fig. 1D) [31]. Finally, analysis of another immune checkpoint inhibitor-treated cohort showed that responding patients had significantly lower *CXCR2* expression than non-responders (Fig. 1E, *p* < 0.05) [32]. These data indicate that *CXCR2* expression correlates with poor therapeutic response in melanoma patients.

### CXCR2 influences tumor differentiation status and enhances tumor growth

To evaluate the role of CXCR2 in *Braf*^*V600E*^*/Pten*^*−/−*^ melanoma tumorigenesis, we crossed C57BL/6 *Tyr-CreER* + *::Braf*^*V600E*^*/Pten*^*fl/fl*^*::mT/mG*:: mice (*Braf/Pten)* [23] with C57BL/6 mice carrying a *Cxcr2*^*fl/fl*^ allele [24] to produce *Tyr-CreER* + *::Braf*^*V600E*^*/Pten*^*fl/fl*^*::mT/mG::Cxcr2*^*−/−*^ (*Braf/Pten/Cxcr2*^*−/−*^) and *Tyr-CreER* + *::Braf*^*V600E*^*/Pten*^*fl/fl*^*::mT/mG::Cxcr2*^*WT*^ (*Braf/Pten/Cxcr2*^*WT*^) littermates. Four-week-old mice were treated with 4-OH tamoxifen (4HT) to induce the tyrosinase promoter-driven Cre-recombinase.

We then utilized flow cytometry to determine whether CXCR2 expression is indeed lost in the tumors that form in the *Braf/Pten/Cxcr2*^*−/−*^ mice. Flow cytometry was first performed on the skin of adult *Braf/Pten/Cxcr2*^*WT*^ and *Braf/Pten/Cxcr2*^*−/−*^ mice immediately after application of 4-HT (prior to tumor formation), and we confirmed that melanocytes do become GFP-positive in both genotypes and that *Braf/Pten/Cxcr2*^*−/−*^ mice lose expression of CXCR2 as expected (Figure S2D). However, after tumor formation, the same assay indicated that ~ 30% of GFP-positive cells in *Braf/Pten/Cxcr2*^*−/−*^ tumors expressed CXCR2. While this is decreased from ~ 70% of GFP-positive cells in *Braf/Pten/Cxcr2*^*WT*^ tumors, is does indicate that CXCR2 positive tumor cells were present during tumor formation in both genotypes (Figure S2E). To confirm these results, we also used immunohistochemistry to co-stain for SOX10 (a melanoma marker) and CXCR2 (Figure S2F). While we do not expect all melanocytic cells in the *Braf/Pten/Cxcr2*^*WT*^ tumors to be CXCR2 positive due to variation caused by cell cycle and differentiation status, we should not see any CXCR2 positivity in the *Braf/Pten/Cxcr2*^*−/−*^ tumor cells. This may be a result of chimerism in the loss of CXCR2 during recombination. It is expected that different floxed alleles recombine at differing efficiencies given the diversity in designs of the floxed alleles and their varied chromatin states. When 2–3 alleles are present in the same cell and all recombine with high efficiency, chimerism is low. However, when recombination efficiency differs between the alleles with one recombining slower than the other, a higher amount of chimerism is expected. Moreover, tamoxifen induction efficiency can also vary and is less efficient than Cre alone [33].

**Fig. 2:**
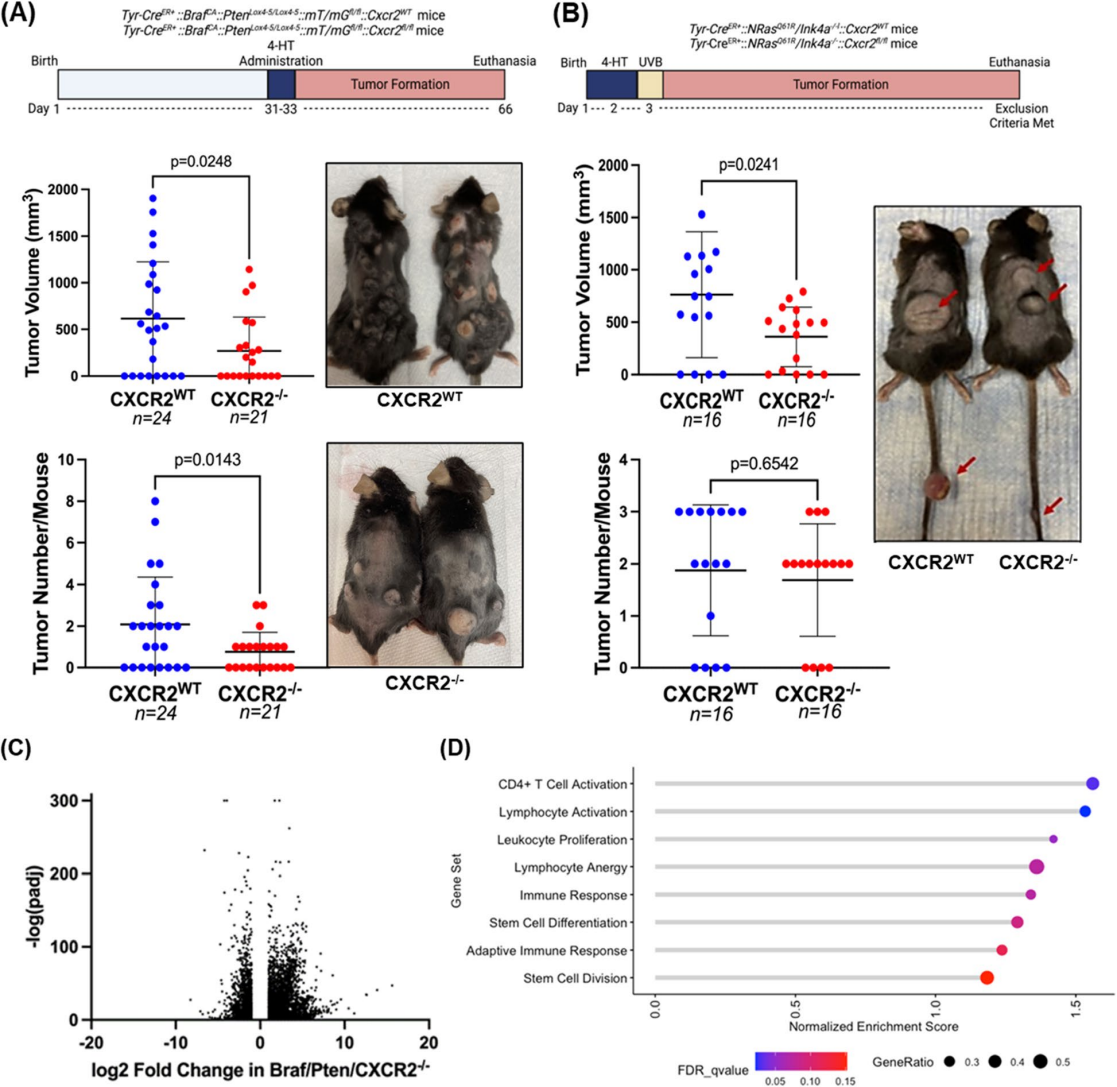
CXCR2 knockout decreases melanoma tumor burden. **a** *Tyr-Cre*^*ER*+^*:: Braf*^*CA/*+^*::Pten*^*lox4−5/lox4−5*^*::mT/mG* C57BL/6 mice were crossed with floxed *Cxcr2* mice to obtain mice with inducible tumors with or without CXCR2 expression. Thirty-six days after 4-HT administration, skin tumor volume and count were recorded, and mice were photographed (significance determined by Welch's t-test). Similarly, **b** *Tyr-Cre*^*ER*+^*::NRas*^*Q61R*^*::Ink4a*^*−/−*^ mice were crossed with floxed *Cxcr2* mice, and resulting pups were treated with 4-HT on days 1 and 2 prior to UV irradiation on day 3 to initiate tumor formation (*n *= 16/genotype). Tumors were measured, counted, and mice were photographed (significance determined by Welch's t-test). RNA was extracted from *Braf*^*V600E*^*/Pten*^*−/−*^*/Cxcr2*^*−/−*^ and *Braf*^*V600E*^*/Pten*^*−/−*^*/Cxcr2*^*WT*^ tumors and subjected to RNAseq analysis. **c** A volcano plot showing fold change and significance of differential gene expression in *Cxcr2*^*−/−*^ tumors compared to *Cxcr2*^*WT*^ tumors. **d** Gene set enrichment analysis (GSEA) of RNAseq data identifies 8 gene sets enriched in *Cxcr2*^*−/−*^ tumors. Point size indicates the gene ratio (percent of genes from the gene set contributing to the enrichment score) and point color represents the FDR q-value

Despite the presence of  approximately 30% CXCR2 positive melanoma cells in *Braf/Pten/Cxcr2*^*−/−*^ tumors, we observed that tumor burden and incidence (Fig. 2A) were significantly reduced in mice with *Braf/Pten/Cxcr2*^*−/−*^ tumors (271 ± 361mm^3^, *n* = 21) compared to mice with *Braf/Pten/Cxcr2*^*WT*^ tumors (615 ± 609mm^3^, *n* = 24, *p* < 0.05) 36 days after 4HT administration. The tumor number per mouse was also reduced upon melanocytic *Cxcr2* deletion (0.7 ± 0.9 vs. 2.1 ± 2.3, *p* < 0.05). These data indicate that CXCR2 signaling plays a role in the induction and growth of *Braf*^*V600E*^*/Pten*^*−/−*^ melanoma.

To determine whether *Cxcr2* is also important in *NRas*^*Q61R*^*/Ink4a*^*−/−*^ melanoma tumors, we crossed *Tyr-CreER* + *::NRas*^*Q61R*^*/Ink4a*^*−/−*^ mice [34] with the *Cxcr2*^*fl/fl*^ mice [24] to produce *Tyr-Cre*^*ER*+^*::NRas*^*Q61R*^*/Ink4a*^*−/−*^*::Cxcr2*^*−/−*^* (NRas/Ink4a/Cxcr2*^*−/−*^*)* and *Tyr-Cre*^*ER*+^*::NRas*^*Q61R*^*/Ink4a*^*−/−*^*::Cxcr2*^*WT*^* (NRas/Ink4a/Cxcr2*^*WT*^*)* littermates. Newborn pups (1–2 days old) were exposed to 4HT, followed by ultraviolet (UV) irradiation on day three, and tumor growth was evaluated over five months. We observed significantly reduced tumor volume with deletion of *Cxcr2* (360 ± 285mm^3^) when compared to *NRas/Ink4a/Cxcr2*^*WT*^ mice (764 ± 601mm^3^) (Fig. 2B, *p* < 0.05, *n* = 16). However, in contrast to the *Braf*^*V600E*^*/Pten*^*−/−*^ model, the number of tumors per mouse was not significantly different between *NRas/Ink4a/Cxcr2*^*−/−*^ (1.69 ± 1.08) and *NRas/Ink4a/Cxcr2*^*WT*^ mice (1.88 ± 1.26, *p* = 0.654). As the *NRas* GEM model requires UV irradiation in addition to the genetic alterations, and the 4HT-induction phase occurs shortly after birth in this model as opposed to 30 days post birth in the BRAF model, it is possible that additional pathways that function independent of Cxcr2 are evoked.

To elucidate the mechanism by which Cxcr2 perturbation in melanocytes could alter the initiation and growth of *Braf*^*V600E*^*/Pten*^*−/−*^* (Braf/Pten)* melanoma, we examined the transcriptome of tumors arising in *Braf/Pten/Cxcr2*^*WT*^ (n = 7) and *Braf/Pten/Cxcr2*^−/−^ (*n* = 8) mice via RNA sequencing (RNAseq) analysis (Figs. 2C, S3A, S4). Interestingly, gene set enrichment analysis revealed that loss of *Cxcr2* expression in *Braf/Pten* tumors resulted in a significant increase in expression of genes involved in CD4 + T cell activation and lymphocyte activation, with a trend toward increased leukocyte proliferation, immune response, and stem cell differentiation (Fig. 2D). However, there is also a paradoxical change in genes involved in lymphocyte anergy. In addition, we see this complex immune modulation reflected in our most differentially expressed genes, with immune-related genes falling into both the most up-regulated and most down-regulated (Figure S3A).

We next utilized the RNAseq data from *Braf/Pten* mice with or without loss of CXCR2 to identify the most differentially expressed genes that are associated with favorable or unfavorable outcome in melanoma patients. We identified the top twenty growth related genes with reduced expression and the top twenty genes associated with inhibition of tumor growth and favorable outcome based on their log_2_ fold change and -log_10_ *p*-value (Figure S4). Key growth stimulatory (Figure S4A) and tumor suppressive genes (Figure S4B) are indicated by red arrows. Genes in common in both enrichment analyses in Figures S3A and S4 include upregulation of the tumor suppressors *Tmprss11e*, *Adamts18* and *Tgm3*, as well as induction of the pyroptosis regulating gene *GSDMc* and the epithelial-specific *Ets* transcription factor 1 (*Elf3*). Commonly down-regulated genes include activators of the lectin pathway of the complement system (*Fcna*), myosin light chain kinase 4 (*Mlk4*), and pathogen recognition receptors (*Cd209*). These changes are plausible contributors to difference in tumor growth observed when* Cxcr2* is targeted in melanocytes during transformation.

### CXCR2 contributes to an immunosuppressive melanoma tumor microenvironment

**Fig. 3:**
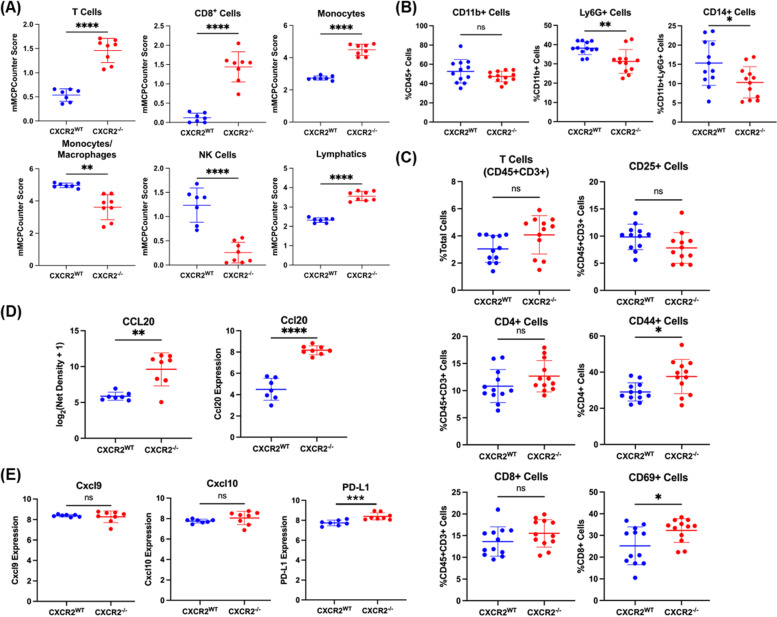
The immune infiltrate of *Braf*^*V600E*^*/Pten*^*−/−*^ tumors is altered with loss of *Cxcr2.* **a** mMCPCounter analysis performed on bulk RNAseq data from *Braf*^*V600E*^*/Pten*^*−/−*^melanoma tumors with or without Cxcr2 predicts significantly enhanced infiltration of T cells, CD8 + T cells, monocytes, NK cells, and lymphatic vessels into *Cxcr2*^*−/−*^ tumors. **b** FACS analysis of CD45 + myeloid cells in *Braf*^*V600E*^*/Pten*^*−/−*^ melanoma reveals decreased MDSC-like cells in *Cxcr2*^*−/−*^ tumors. **c** FACS analysis of CD45 + cells in *Braf*^*V600E*^*/Pten*^*−/−*^ melanoma tumors identified changes in activated CD4 + CD44 + T cells and CD8 + CD69 + T cells. **d** Cytokine array for 62 cytokines expressed in TME of *Braf*^*V600E*^*/Pten*^*−/−*^ tumors revealed one major cytokine, CCL20, that is strongly upregulated with loss of *Cxcr2* (*n* = 4/genotype) based on net density. These data are complemented by increased *Ccl20* mRNA with loss of *Cxcr2* in *Braf*^*V600E*^*/Pten*^*−/−*^ tumors. **e** *Cxcl9, Cxcl10*, and *PD-L1* expression based upon RNAseq analysis from *Braf*^*V600E*^*/Pten*^*−/−*^ tumors expressing or not expressing Cxcr2 in melanocytes. All statistical significance determined via Welch’s t-test

Due to the GSEA-indicated enrichment in gene sets associated with CD4 + T cell activation, lymphocyte activation, and leukocyte proliferation in *Braf/Pten/Cxcr2*^*−/−*^ tumors (Fig. 2D), we then evaluated the immune cell infiltrate between *Braf/Pten/Cxcr2*^*WT*^ and *Braf/Pten/Cxcr2*^*−/−*^ tumor-bearing mice. We first utilized the murine Microenvironment Cell Population counter (mMCPcounter) [34], an immune deconvolution algorithm developed for bulk murine RNAseq data. mMCPcounter predicted an increase in CD3 + T cells, CD8 + T cells, monocytes, lymphatic vessels, and eosinophils, as well as a decrease in mast cells, NK cells, and endothelial cells (*p* < 0.05) (Figs. 3A, S5A), suggesting enhanced anti-tumor immunity in the *Braf/Pten/Cxcr2*^*−/−*^ TME. To analyze the immune environment in vivo, we defined the profile of CD45 + cells from *Braf/Pten/Cxcr2*^*WT*^ and *Braf/Pten/Cxcr2*^−/−^ tumor-bearing mice using FACS analysis. In agreement with the mMCPcounter predicted leukocytic infiltrates, we observed that deletion of* Cxcr2* in melanocytes undergoing transformation skewed the TME toward anti-tumor immunity. FACS analysis of the CD45 + cells in *Braf/Pten/Cxcr2*^−/−^ tumors revealed a decrease in the immunosuppressive Ly6G + CD11b + (*p *< 0.01) and CD14 + G-MDSC (*p* < 0.05) cells, with no change in total CD11b + cells (Fig. 3B), in addition to a trend toward decreased CD25^hi^CD45 + CD3 + regulatory T cells and a trend toward an increase in the frequency of CD3 + CD8 + T cells. There was also a significant increase in memory CD44 + CD4 + T cells (*p* < 0.05) and activated CD69 + CD8 + T cells (*p* < 0.05) within the *Braf/Pten/Cxcr2*^−/−^ tumors (Fig. 3C, S5D). We also validated these results with immunohistochemical staining of tumor sections (Figure S6A). FACS analysis of peripheral blood cells revealed no significant difference in any immune population between *Braf/Pten/Cxcr2*^*−/−*^ mice and *Braf/Pten/Cxcr2*^*WT*^ mice before or after tumor formation. (Figure S5B, S6B).

The identified differences in immune cell infiltrate are highly suggestive of altered cytokine signaling within the TME. Therefore, a 62-cytokine array was performed on *Braf/Pten/Cxcr2*^*WT*^ (*n* = 4) and *Braf/Pten/Cxcr2*^*−/−*^ (*n* = 4) tumor lysates. CCL20, an inflammatory chemokine that is highly chemotactic for CCR6-expressing lymphocytes and dendritic cells, is strongly upregulated (24-fold) in the *Braf/Pten/Cxcr2*^*−/−*^ TME (Fig. 3D). In addition, RNAseq analysis revealed a significant increase in PD-L1 expression in tumors from *Braf/Pten/Cxcr2*^*−/−*^ mice compared to *Braf/Pten/Cxcr2*^*WT*^ mice (Fig. 3E). Furthermore, M-CSF, eotaxin, and MIP-2 were slightly increased, which could contribute to myeloid cell infiltration, and there was a slight decrease in IL-1β in the tumors from *Braf/Pten/Cxcr2*^*−/−*^ mice as compared to tumors from *Braf/Pten/Cxcr2*^*WT*^ mice (Figure S5C). These data suggest that targeted deletion of *Cxcr2* in melanocytes during tumorigenesis results in a marked increase in CCL20 and additional subtle changes in the cytokine milieu of the TME.

### CXCR1/CXCR2 antagonist SX-682 inhibits *Braf*^*V600E*^*/Pten*^*−/−*^ and* NRas*^*Q61R*^*/Ink4a*^*−/−*^ melanoma tumor growth and promotes anti-tumor immunity

**Fig. 4:**
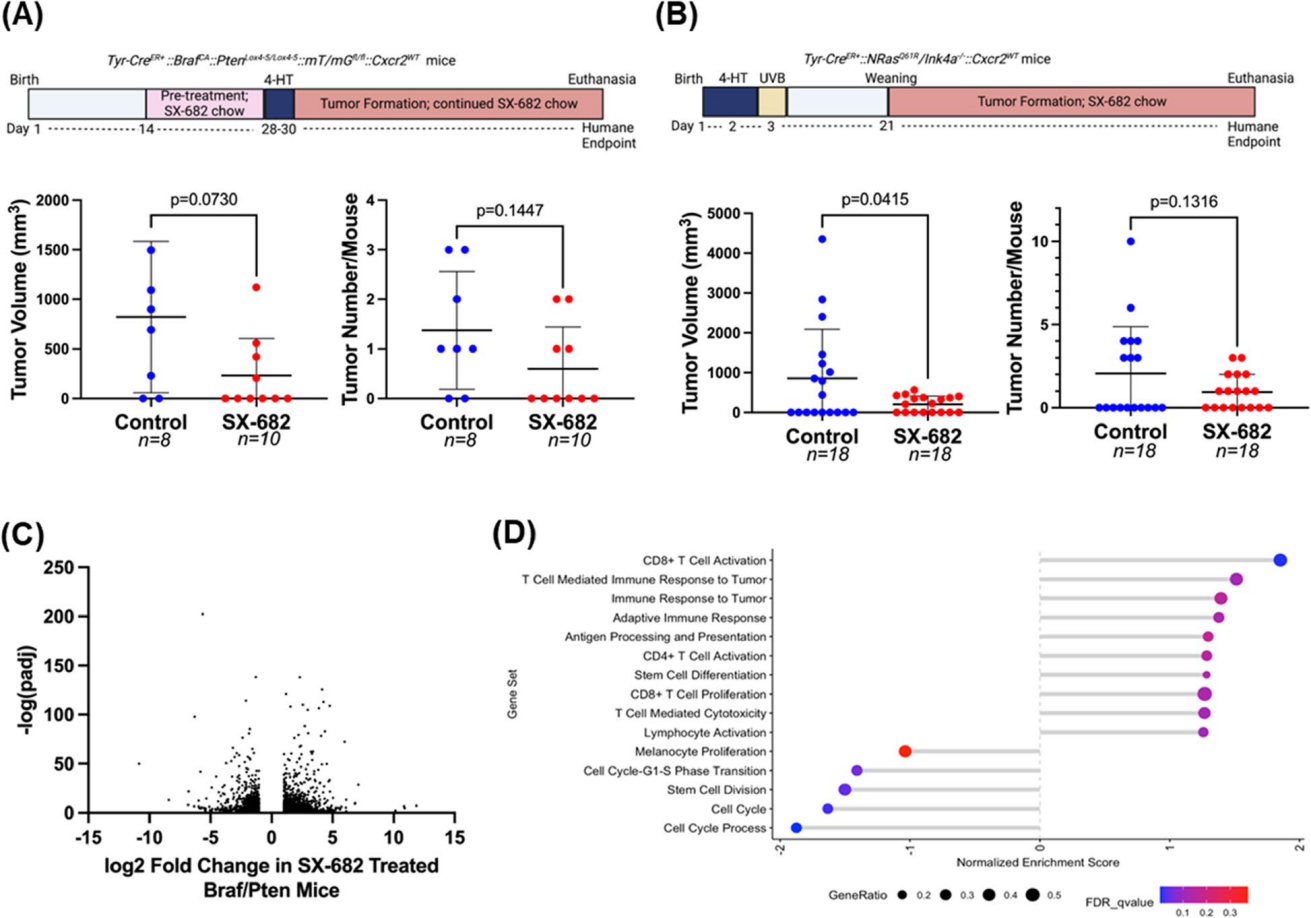
SX-682 affects *Braf*^*V600E*^*/Pten*^*−/−*^ and *NRas*^*Q61R*^*/Ink4a*^*−/−*^ tumorigenesis. **a** *Braf*^*V600E*^*/Pten*^*−/−*^ and **b** *NRas*^*Q61R*^*/Ink4a*^*−/−*^ mice were fed chow containing SX-682 or vehicle continuously through tumor formation, and tumors were measured and counted. Significance was determined using a Welch's t-test. **c** A volcano plot showing fold change and significance of differential gene expression between tumors from SX-682-fed and control-fed *Braf*^*V600E*^*/Pten*^*−/−*^ mice. **d** Gene set enrichment analysis of SX-682 treated or control *Braf*^*V600E*^*/Pten*.^*−/−*^ tumors identifies gene sets enriched in SX-682 treated tumors (positive normalized enrichment score) or enriched in control tumors (negative normalized enrichment score)

Having established the importance of *Cxcr2* in the development, growth, and TME of *Braf/Pten* melanoma tumors, we sought to evaluate the therapeutic potential of systemic CXCR1/CXCR2 inhibition. Thus, chow containing the CXCR1/CXCR2 antagonist SX-682 [35] was administered to four-week-old mice. After two weeks of eating vehicle control or SX-682-containing chow, 4-HT was applied to the backs of the mice for three successive days. Following a month of continuous feeding on control or SX-682-containing chow, we observed that *Braf/Pten* mice fed SX-682-containing chow exhibited a trend toward reduction in tumor volume compared to mice fed vehicle control chow (Fig. 4A, *p* = 0.07; 802.5 ± 724.01mm^3^ for control; 230.20 ± 373.21 mm^3^ for SX-682). Moreover, there was a trend toward decreased tumor formation in SX-682-fed mice (*p* = 0.145), where only 40% (4/10) of SX-682-fed mice developed tumors compared to 75% (6/8) of control-fed mice (Fig. 4A). Similarly, *NRas*^*Q61R*^*/Ink4a*^*−/−*^ (*NRas/Ink4a)* mice were fed chow containing SX-682 or control chow, and tumors that developed over five months were counted and measured. We observed that SX-682 treatment significantly suppressed tumor growth (*p* = 0.041, Fig. 4B) but only trended toward a decrease in tumor incidence (*p* = 0.111, Fig. 4B). Overall, SX-682 produced inhibition of tumor volume comparable to that of CXCR2 loss in melanocytes but did not impact tumor formation as significantly. This suggests that at our current dosage of SX-682 in the chow, we are unable to achieve complete suppression of CXCR2 at the time of tumor initiation.

RNA sequencing analysis of control and SX-682 treated tumors from *Braf/Pten* mice identified nearly 3000 differentially expressed genes with many trends similar to those observed in *Braf/Pten/Cxcr2*^*−/−*^ tumors. A volcano plot shows that a significant number of genes were strongly up or down-regulated (log_2_ fold change of > 3) with a very high level of significance (-log_10_(P-adj) > 50) (Fig. 4C). Upregulated genes include those involved in regulation of growth, proliferation, and cell cycle*;* tumor suppression*;* differentiation/stemness; immune regulation; and motility and adhesion. Genes downregulated in response to Cxcr1/Cxcr2 antagonism with SX-682 include those involved in cell adhesion and cell proliferation, cell cycle, and growth (Figure S3B).

**Fig. 5:**
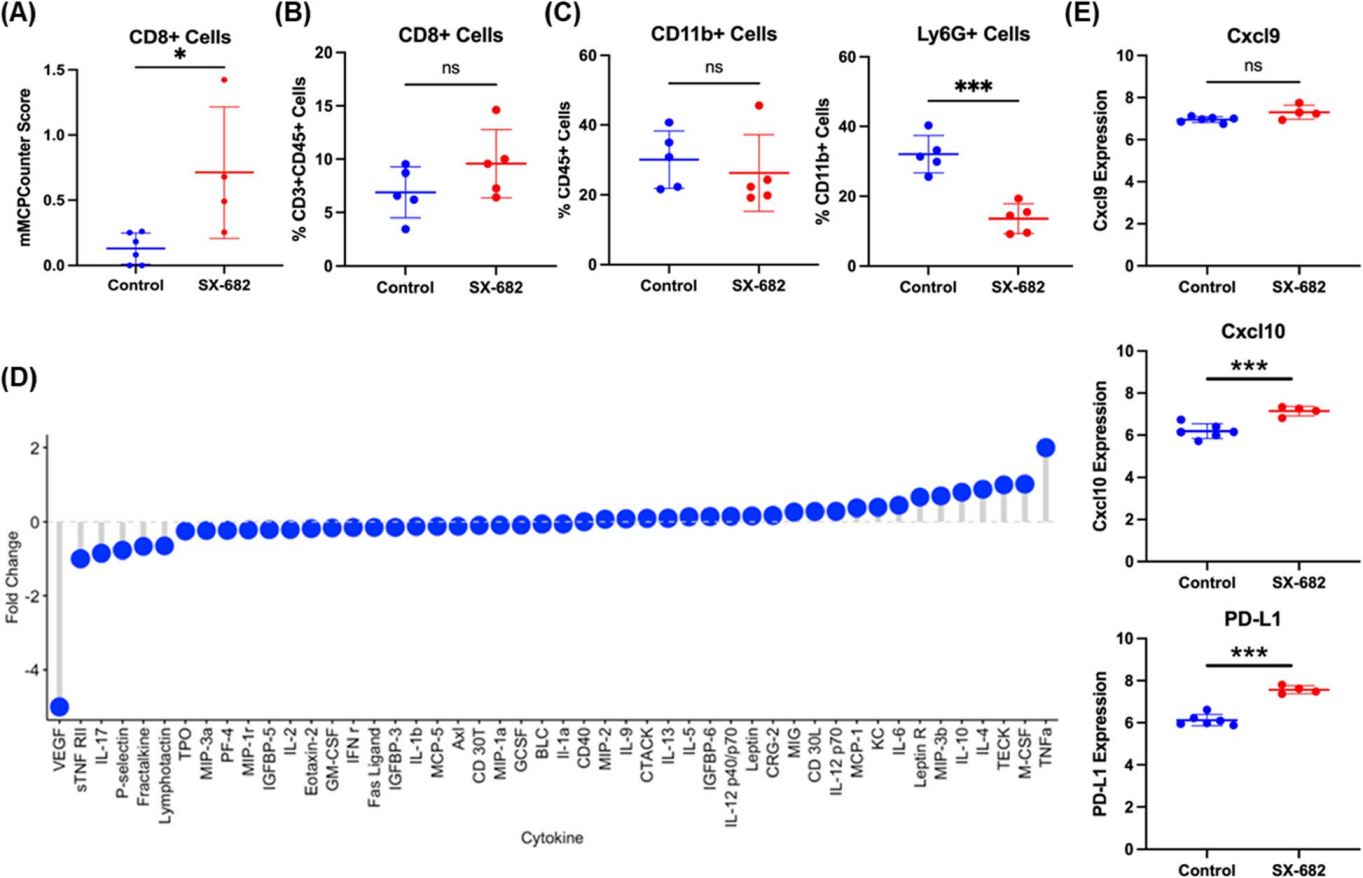
SX-682 alters the immune profile of *Braf*^*V600E*^*/Pten*^*−/−*^ melanoma. **a** mMCPCounter analysis of bulk RNAseq data predicts enrichment for CD8 + T cell infiltrate into tumors following treatment with SX-682 (*p* < 0.05). **b** FACS analysis confirms a trend toward increased CD8 + T cells in SX-682 treated *Braf*^*V600E*^*/Pten*^*−/−*^ melanoma. **c** FACS analysis of CD45 + myeloid cells indicated a significant decrease in immunosuppressive CD11b + Ly6G + cells, but no change in total CD11b + cells. **d** A cytokine array was performed on control and SX-682 treated tumors, identifying a notable decrease in Vegf and an increase in Tnfα. **e** *Cxcl9, Cxcl10,* and *Pd-l1* expression based upon RNAseq analysis from SX-682 or control treated tumors. All statistical significance determined via Welch’s t-test

GSEA of the tumors from *Braf/Pten* mice treated with SX-682 revealed a significant increase in CD8 + T cell activation, with trends toward increased T cell-mediated immune response to the tumor, immune response to tumor, adaptive immune response, antigen processing and presentation, CD4 + T cell activation, stem cell differentiation, CD8 + T cell proliferation, T cell-mediated cytotoxicity, and lymphocyte activation. There were significant decreases in genes involved in melanocyte proliferation, cell cycle process, cell cycle, stem cell division, and cell cycle G1-S transition (Fig. 4D). mMCPcounter analysis of the tumor RNAseq data predicted an increase in CD8 + T cells (Fig. 5A) and monocytes (Figure S7A), and a decrease in B-derived cells and cells of the lymphatics (*p* < 0.01) in tumors from the SX-682-treated *Braf/Pten* mice (Figure S7A).

FACS analysis of SX-682 treated *Braf/Pten* tumors revealed a trend toward increased CD8 + T cells (*p* = 0.17), no change in CD11b + cells, and a significant decrease in CD11b + Ly6G + cells (*p* < 0.001) (Fig. 5B, C). Additional FACS analysis of tumor CD45 + cells showed a decrease in CD4 + CD3 + cells (*p* < 0.05) in tumors from the SX-682 chow-fed mice (Figure S7C). In peripheral blood, there was a significant decrease in CD44 + CD4 + T cells and CD62L + CD4 + T cells and a trend toward increased CD69 + CD8 + T cells from mice fed SX-682 chow (*p* = 0.059; Figure S7B). In addition, a cytokine array of tumor lysates (*n* = 4 for each genotype) revealed a marked reduction in VEGF, indicating a reduction in tumor angiogenesis, and an increase in TNFα, indicating a more inflammatory tumor microenvironment (Fig. 5D). Moreover, RNAseq analysis of *Braf/Pten* tumors revealed that SX-682 induces expression of *Cxcl9, Cxcl10*, and *Pd-l1* (Fig. 5E). Altogether, these data indicate that SX-682 alters the TME to stimulate anti-tumor immunity and reduce tumor growth.

When we evaluated the hematological effects of SX-682 in a rodent model during the IND-enabling toxicology assessment, we saw reversible neutropenia following treatment with no significant effect on other peripheral blood components (Figure S7D). These data indicate that SX-682 more widely affects the immune cell population in tumor bearing as compared to tumor free rodents. We also evaluated the effects of short term SX-682 treatment on normal mice. C56BL/6 mice were treated with 50 mg/kg SX-682 daily via oral gavage for 4 days. The peripheral blood leukocytes were analyzed by FACS, and we found that SX-682 reduced the percentage of Ly6G + cells that were CD14 + (*p* = 0.04) and increased the percentage of CD45 + cells that were CD19 + (*p* = 0.026) (Figure S7E).

### SX-682 treatment of Melan-A, B16F0, and B16F10 cells reveals tumor cell-specific gene modulation

**Fig. 6:**
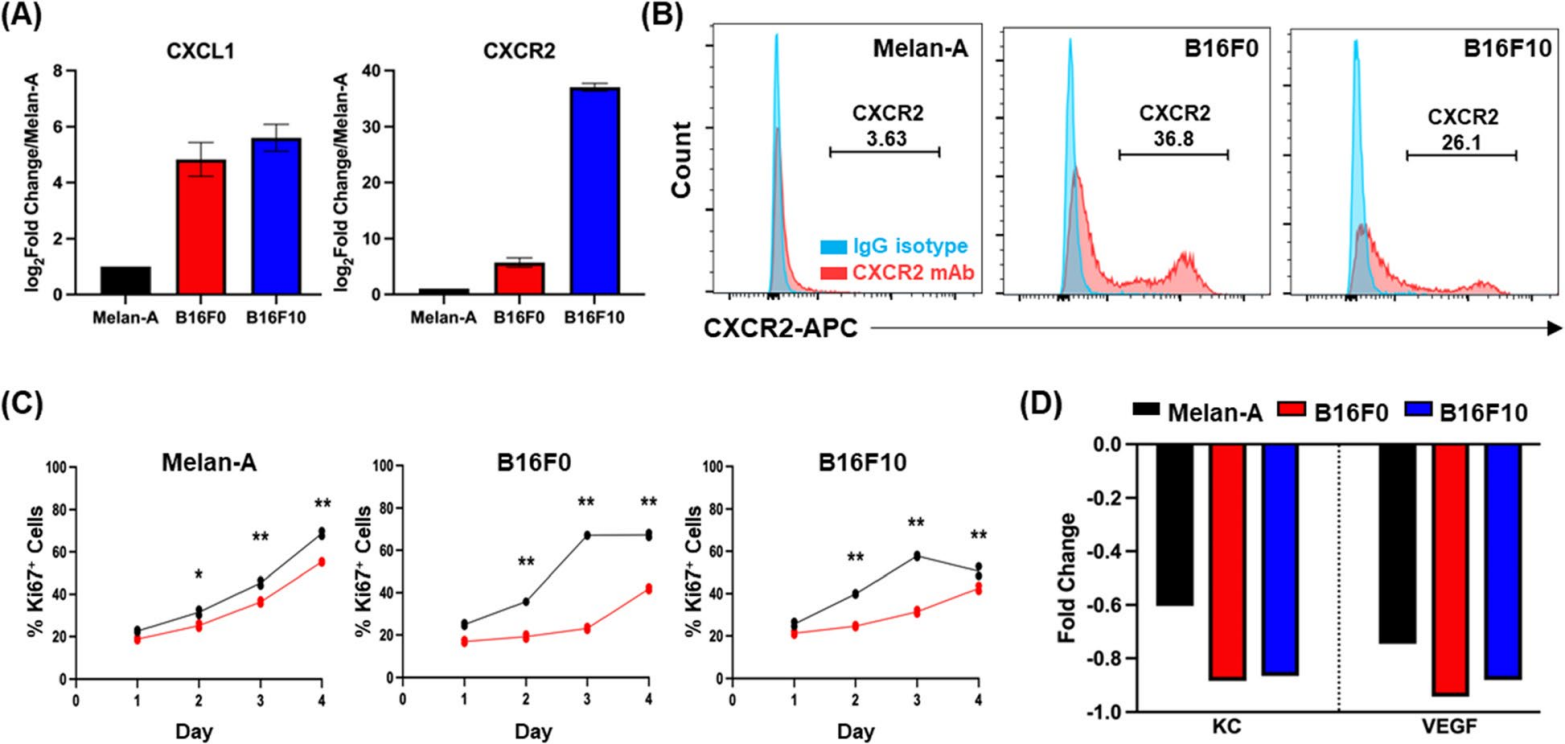
Tumor cell-specific impacts of SX-682. CXCL1 and CXCR2 expression on Melan-A, B16F0, and B16F10 cells based on **a** the NCBI database and **b** CXCR2 expression in Melan-A, B16F0 and B16F10 cells based on flow cytometry. **c** Cell lines were treated with 5 µM SX-682 (or DMSO control) for 4 days prior to staining with Pacific Blue-Ki67 for FACS analysis. The percentage of positive staining cells was significantly decreased in the SX-682 treated cells for all cell lines (analyzed using a two-way ANOVA with Benjamini and Hochberg (BH) correction for multiple tests). **d** Cytokine array of SX-682-treated Melan-A, B16F0 and B16F10 cells shows that SX-682 treatment reduced the expression of KC and VEGF in all three cell lines

Our murine experiments involved bulk RNA sequencing of tumors that contain tumor cells in addition to stromal and immune cells. To identify the specific effect of SX-682 treatment on tumors without the contribution of other cell types, we investigated the effect of SX-682 on non-tumorigenic Melan-A cells, tumorigenic B16F0 cells, and metastatic B16F10 cells in vitro. First, we evaluated Cxcr2 expression and found that B16F0 and B16F10 cells express significantly more Cxcr2 than Melan-A cells, as evaluated by mRNA levels and surface protein labeling (Fig. 6A, B). We then analyzed the effect of SX-682 (5 μM) on the growth of these cells and observed that SX-682 treatment resulted in a small but significant inhibition of growth in B16F0 and B16F10 cells in vitro based on the percentage of cells staining positively for Ki-67 (Fig. 6C) and cell number (Figure S8A). In addition, SX-682 treatment of B16F0 and B16F10 cells in vitro also reduced production of both Cxcl1 (KC) and vascular endothelial growth factor (Vegf) as evaluated by cytokine array (Fig. 6D), again indicating the potential for SX-682 to impact the immune profile of the TME.

To identify tumor cell-specific transcriptional changes following SX-682 treatment, we performed bulk RNAseq analysis on each of the three cell lines. Of the total differentially expressed genes, expression of 4024 genes was altered in all three lines. An additional 860 genes were differentially expressed in both tumorigenic B16F0 and B16F10 lines in response to SX-682 (Figure S3C and 7A ). Commonly upregulated genes include those involved in apoptosis and cell stress response and suppression of gluconeogenesis. In contrast, commonly down-regulated genes include those involved in methylation, RNA splicing, and cell cycle processes (Figure S3C and S8B). Reverse phosphoprotein analysis (RPPA) identified SX-682-induced decreases in phosphoproteins involved in growth (AKT, BRAF, pS445-BRAF, CDC2-pY15, CDC6, GSK-3b, mTOR, mTOR pS2448, MMP14, PAX8, and S6), as well as SX-682-induced increases in immunomodulatory proteins (STING, PD-1, PD-L1, TRIM25, and ANNEXIN I); proteins involved in the regulation of apoptosis (PUMA, BLC2, BLC2A1, BCLxL, Smac); tumor suppressors (TSC2, WTAP); and cell cycle regulators (CDC25, CDC42, PLK1, EGFR, PRAS40_pT246). Of interest, β-catenin expression is increased following SX-682 treatment. This is counter-intuitive for SX-682 inhibition of tumor growth, as the Wnt/β-catenin pathway often drives melanoma tumor growth and metastasis. However, we observed that the phosphorylated forms of β-catenin (pT41 and pS45) that enable its ubiquitin-mediated degradation are increased as well. This indicates that β-CATENIN is marked for degradation, thus diminishing the potential for enhanced tumor growth. There were also increases in proteins involved in motility: MYOSIN-Iia, PAK, CDC-42, MYOSIN Iia-pS1943, and HMGA1 (Figure S8C, D). Finally, there were only subtle changes in cytokine expression in response to SX-682 treatment in vitro*,* and these were inconsistent across the three cell lines (Figure S8E). Altogether, these results suggest that multiple compounding signals are induced in cells treated with SX-682, including a decrease in growth signaling, modulation of apoptosis, enhanced anti-tumor immunity, and altered cell cycle processes.

### *Tfcp2l1* distinguishes the Cxcr2^WT^ from the Cxcr2 perturbed phenotype

**Fig. 7:**
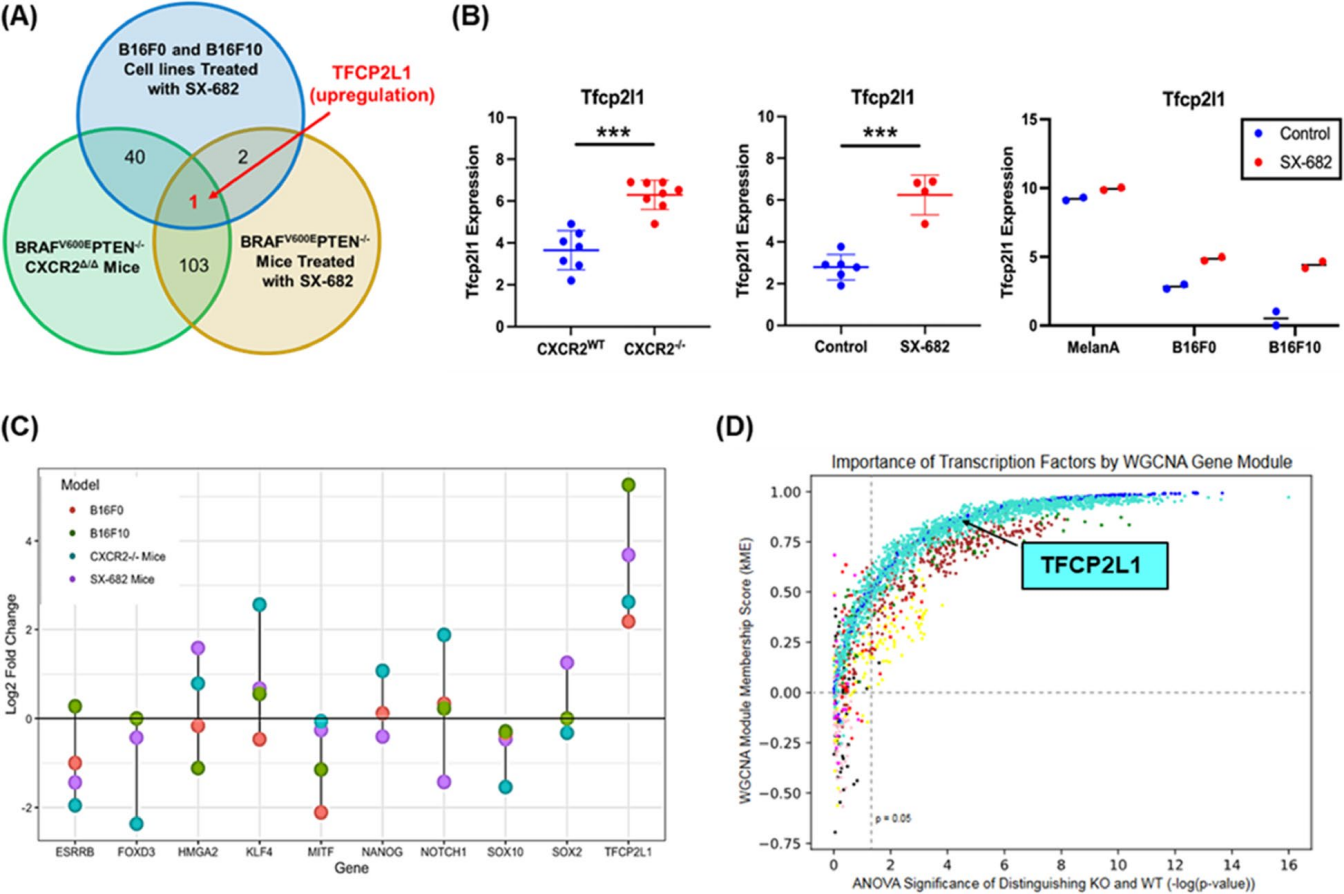
*Tfcp2l1* is commonly upregulated across three models of CXCR2 perturbation. **a**,** b** In comparing expression data from *Braf*^*V600E*^*/Pten*^*−/−*^*/Cxcr2*^*−/−*^ tumors. *Braf*^*V600E*^*/Pten*^*−/−*^ tumors treated with SX-682, and B16F0 and B16F10 cell lines treated with SX-682, *Tfcp2l1* was consistently upregulated compared to appropriate controls (as determined by Welch's t-test). **c** Log_2_ fold change for *Tfcp2l1* and related genes across experimental groups based upon RNAseq analysis. **d** Identification of transcription factors central to Weighted Correlation Network Analysis (WGCNA) co-expressed gene modules (by kME) and significantly differentially expressed between *Braf*^*V600E*^*/Pten*^*−/−*^*/Cxcr2*^*−/−*^ and *Braf*^*V600E*^*/Pten*^*−/−*^*/Cxcr2*^*WT*^ tumors. TFs are colored by gene module and show varying levels of centrality to each module and importance in distinguishing WT and KO tumors. Turquoise dots represent transcription factors that are up in the *Braf*^*V600E*^*/Pten*^*−/−*^*/Cxcr2*^*−/−*^ tumors and blue dots represent transcription factors that are up in the *Braf*^*V600E*^*/Pten*^*−/−*^*/Cxcr2*^*WT*^ tumors

To better understand the complex transcriptional reprogramming that occurs when CXCR2 activity is diminished via knockout or with SX-682 treatment, we compared differentially expressed genes in *Braf/Pten/Cxcr2*^*−/−*^ tumors, SX-682-treated tumors, and SX-682-treated tumorigenic B16F0 and B16F10 cell lines compared to controls. We noted that based upon our search for genes with a minimum of a log_2_ fold change > 2 and a *p*-value < 0.05, only one gene stood out as significantly upregulated across all four models compared to the respective controls: Transcription factor CP2 like-1(*Tfcp2l1)* (Fig. 7A, B). To verify the RNA sequencing results, we performed RT-PCR analysis of RNA samples from MelanA, B16F0, and B16F10 cells to determine *Tfcp2l1* expression. With this assay, we show that SX-682-treatment elevates *Tfcp2l1* expression in the tumorigenic cell lines (Figure S10).

TFCP2L1 is a member of the TFCP2/TFCP2L1/UBP1 subfamily of transcription factors that contributes to the maintenance of stemness in pluripotent stem cells and can also exhibit tumor suppressive activity and modulate differentiation [26, 36–39]. The Krupple-like Factor (KLF) family of transcription factors works with and can be induced by Tfcp2l1 to modulate induction and maintenance of naïve pluripotency in mouse primordial germ cells [40–42]. It has been previously reported that *TFCP2L1* is positively associated with expression of pluripotency genes including *Nanog, Oct4, Sox2,* and *Esrrb* in mouse embryonic stem cells [42]. However, our data suggest a complex relationship between Cxcr2 perturbation and Tfcp2l1-related gene expression. In the *Braf/Pten* model, stemness marker *Esrrb* and neural crest markers *Foxd3* and *Sox10* were decreased when *Cxcr2* was deleted in tyrosinase expressing cells. In contrast, stemness markers *Tfcp2l1*, *Klf4* and *Hmga2,* were increased. In SX-682 treated *Braf/Pten* model, there was a trend toward a decrease in stemness marker *Esrrb,* a significant decrease in the neural crest marker *Sox10*, and a small but significant decrease in the melanoblast marker *Mitf* (Figs. 7C, S8A-L). The melanocyte differentiation marker *Tyr* was increased in both the CXCR2^−/−^ and the SX-682 treated *Braf/Pten* mouse models. In the B16F0 and B16F10 cells, RT-PCR analysis revealed that stemness markers *Esrrb, Hmga2, Myc, Sox2*, neural crest marker *Sox10,* and melanoblast marker *Mitf* were significantly decreased in response to SX-682-treatment in vitro. *Foxd3* was significantly decreased in B16F10 and trended toward significant reduction in B16F10. In contrast, stemness markers *Tfcp2l1, Nanog* and *Notch1* were increased, while there was a decrease in *Tyr* expression (Figure S10)*.* Altogether, these data imply that with ablation of Cxcr2 activity, there is an increase in some stemness markers but a decrease in others, a decrease in neural crest markers, and a trend toward a decrease in melanoblast markers. However, there is variability in the mix of these markers from model to model. In the mouse models, tyrosinase (*Tyr*) continues to be highly expressed, though in the B16 cell cultures, SX-682 decreased its expression. Thus, it is not clear that upregulation of TFCP2L1 is associated with an increase in a population of pluripotent cells in these melanoma tumors. However, we cannot rule out the possibility that overtime there was a shift in the differentiation pattern of a population of pluripotent cells toward a mesenchymal phenotype.

To support the relevance of upregulation of the transcription factor, *Tcfp2l1*, in association with loss of CXCR2 signaling, we performed an orthogonal approach based on weighted gene co-expression network analysis (WGCNA). WGCNA was applied to the RNA-seq data from these tumors to generate groups of highly correlated genes, or gene modules, that functionally distinguish *Braf/Pten/Cxcr2*^*−/−*^ and *Braf/Pten/Cxcr2*^*WT*^ tumors (Figure S11). Using an ANOVA test between sample conditions, we found six distinct modules that significantly distinguish the transcriptional programs of *Cxcr2*^*WT*^ and *Cxcr2*^*−/−*^ tumors (Figure S11). Gene ontology (GO) analysis showed each module is enriched in distinct functions: the *Cxcr2*^*WT*^-upregulated modules are enriched in GO terms such as protein localization to mitochondrion (blue), aerobic respiration and oxidative phosphorylation (green and brown, respectively), and signaling (yellow), while the *Cxcr2*^*−/−*^-upregulated modules are enriched for GPCR signaling (red) and skin development (turquoise). These changes in gene expression may result as an adaptation to the loss of CXCR2 function. Interestingly, the WGCNA module membership score (kME) indicated that Tfcp2l1 is central to the turquoise module (kME = 0.854) involved in skin development and is significantly upregulated in the *Cxcr2*^*−/−*^ samples (FDR-adjusted *p* = 0.0000286) (Fig. 7D).

Finally, to define the activity of TFCP2L1 following CXCR2 perturbation, we performed chromatin immunoprecipitation and sequencing analysis (CHIPseq) on B16F0 tumorigenic melanoma cells following treatment with vehicle or SX-682. In identifying promoters bound by Tfcp2l1 in each condition in addition to RNAseq data, we can delineate SX-682-induced gene set enrichment. Interestingly, enrichment analysis of TFCP2L1-bound genes revealed that SX-682 treatment increased expression of genes associated with the adaptive immune system and response to hormones (Figure S12A). SX-682 treatment also enriched Tfcp2l1 binding to and repression of genes involved in the RAF/MAPK cascade, β-catenin independent Wnt signaling pathways, and catabolism (Figure S12B). When data from RNAseq, RPPA, and ChIPseq analysis were examined using Metascape, key regulatory pathways emerged as commonly associated with CXCR2 loss of function (Figure S12C, D). These data are consistent with the observed reduction in tumor growth when CXCR2 signaling is blocked and suggest that changes in gene expression are associated with Tfcp2l1 transcriptional control.

### Does CXCL1 activation of normal melanocytes suppress the TFCP2L1 transcriptional program?

To gain insight into how CXCL1 activation of CXCR2 regulates the expression of stemness and differentiation markers, RNAseq analysis was performed on normal human epidermal melanocyte (NHEM) cultures treated with CXCL1 or with CXCL1 and SX-682 (Figure S13). CXCL1 supplementation was utilized in this model to recreate the enhanced baseline CXCR2 activation of tumorigenic cells. Consistent with this, CXCL1 increased the proliferation of NHEM cells in vitro (Figure S13A). Moreover, CXCL1 treatment increased expression of a number of genes, and this effect was lost with SX-682 treatment (Figure S13B). For example, CXCL1 treatment of NHEM cells induced a trend toward increased expression of *MITF, BMP6, WNT5A, and SOX10*, and the addition of SX-682 reversed this trend. Moreover SX-682 treatment resulted in induction of expression of a host of genes that are lowly expressed in control and CXCL1 treated NHEM cells (Figure S13B) and suppresses expression of many highly expressed genes in control and SX-682-treated NHEMs (Figure S13B). SX-682 also induced a trend toward elevated *TFCP2L1, KLF4, FOXD3, FOXD1*, and *CCL20* expression over that produced by CXCL1 alone (Figure S13C). These data clearly show that loss of CXCR2 activity dramatically alters gene expression, resulting in reduced CXCL1-induced proliferation of NHEM. In addition to the effects on stemness and differentiation markers, we also found that several chemokines, interleukins (Table S1), and TNF-related cytokines and interferons (Table S2) were altered when NHEMs were treated with combined CXCL1 and SX-682. SX-682 treatment increased expression of inflammatory genes *CCL20, IL18R1, IL1RL1* and decreased expression of chemokines associated with macrophage and MDSC recruitment (*CCL2, CCL7, CCL8, CXCL1, CXCL12, CXCL6* and *IL33)* as well as TNF family members involved in MAPK activation, osteoclastogenesis, and B cell activation (*C1QTNF2, TNFRSF21, TNFSF11*, and *TNFSF13B).*

Taken together, our data from both tumor models and in vitro studies show that CXCR2 activation is associated with activation of the MAPK cascade, AKT, and WNT signaling, expression of chemokines that recruit MDSCs and pro-tumor macrophages, and enhanced tumor growth. In contrast, loss of CXCR2 or inhibition of CXCR1/CXCR2 in melanoma progenitor cells is associated with expression of genes associated with inflammation, T cell recruitment, pluripotency, and reduced tumorigenicity. The mechanism for these changes in gene expression is in part due to induction of *Tfcp2l1*, a member of the TFCP2/TFCP2L1/UBP1 transcription factor subfamily that can serve as a pro-oncogenic factor in some tumors or in the case of melanoma, a tumor suppressor [26, 43].

## Discussion

The CXCR1 and CXCR2 receptors are G protein-coupled receptors that generate downstream signals including PI3K and AKT, often implicated in growth [6, 11, 44–46]. The role of CXCR2 in cell motility has been well characterized, and the signals generated through this receptor leading to activation of AKT and ERK also modulate cell proliferation and growth [47, 48].

CXCR1 has been reported to be important for the renewal of a population of stem cell-like cells in human breast cancer [49]. In mice, CXCR2 controls functions normally regulated by CXCR1 in humans, thus it is plausible that CXCR2 may also modulate stemness. While shRNAs have previously been used to knock down *CXCR1* and *CXCR2* in a human melanoma cell line showing inhibition of tumor growth and microvessel density, these experiments were performed in immune deficient mice and with only one cell line [50]. Here, we examined the role of CXCR2 in melanocyte tumorigenesis and observed that targeted deletion of CXCR2 in tyrosinase-expressing melanocytes reduced melanoma tumor burden in *Braf/Pten and NRas/Ink4a* murine melanoma and modulated the expression of melanocyte stemness and differentiation markers, despite the presence of chimerism in our knockout tumors.

We observed that the mechanism by which loss of Cxcr2 activity during melanocyte tumorigenesis resulted in reduced tumor growth in *Braf/Pten* mice was due to major changes in gene expression, with decreased expression of genes involved in proliferation and increased expression of genes associated with tumor suppression, T cell recruitment and differentiation, and apoptosis. These gene expression data from RNAseq analysis were further supported by phospho-proteomic data. We observed that loss of Cxcr2 activity in tumor cells resulted in a change in the tumor immune microenvironment, with increased CD8 + T cells and reduced macrophages and MDSC-like cells. When Cxcr1/Cxcr2 were antagonized in *Braf/Pten* mice and tumorigenic melanoma cell lines via treatment with SX-682, similar alterations in tumor growth and the gene expression profiles were achieved, and this was accompanied by development of anti-tumor immune microenvironment. While our experiments were designed to determine the effects of SX-682 on tumor formation and growth, other studies have shown that when SX-682 is administered after tumors have formed, it can significantly inhibit the growth of preformed tumors as a single agent (Supplementary Table 3).

When we looked for genes significantly induced in Cxcr2^−/−^ tumors, SX-682 treated tumors, and B16F0 and B16F10 cell lines, one common gene emerged: *Tfcp2l1*. *TFCP2L1* is a crucial transcription factor that induces the expression of genes associated with stemness in embryonic stem cells [26]. As such, we probed the relationship between Tfcp2l1, differentiation along the melanocyte lineage, and cancer stem cells within melanoma.

Much of our understanding of melanocyte lineage came from in vitro studies that involved the differentiation of human pluripotent stem cells along a neural crest lineage, then on to form melanocytes [51]. Wnt ligands and Bmp4 induce the early transition of Oct4 + Nanog + pluripotential cells into Sox10 + neural crest cells. Exposure to endothelins and BMP4 promotes neural crest cell differentiation to M+ cKIT + melanoblasts, and these can be terminally differentiated to TYR + OCA2 + melanocytes through continued exposure to WNT ligands, BMP4, and induction of intracellular cAMP [52]. In the melanoma models used in our studies, the targeted alterations in gene expression (*Braf/Pten/Cxcr2*^*−/−*^) occur in tyrosinase expressing melanocytes. Interestingly, while loss of CXCR2 expression or activity was not associated with reduction in tyrosinase in our mouse models, we noted a decrease in the expression of neural crest markers *Sox10* and *Foxd3* in tumors that developed when Cxcr2 activity was ablated. However, IHC analysis of SOX10 did not reflect a change in the protein levels of SOX10 in tumors that developed *Braf/Pten/Cxcr2*^*−/−*^ mice as compared to *Braf/Pten/Cxcr2*^*WT*^ mice*.* In addition, there was an increase in expression of some markers associated with pluripotency or stemness.

While we do see consistent *Tfcp2l1* induction across all our models of Cxcr2 perturbation, trends in TFCP2L1-regulated genes are not as clear. There is a trend toward increased *Klf4, Hmga2, Notch1, Myc*, and *Stat3* expression which would suggest that tumors with loss of* Cxcr2* are less differentiated. However, Esrrb, which has been established as a direct target of TFCP2L1 binding and induction in ESCs [42], is significantly decreased in our *Cxcr2*^*−/−*^ tumors. The implications of this shift in stemness markers in relation to melanoma aggression, treatment sensitivity, and overall prognosis is currently unknown. However, it has been previously demonstrated that CXCR2 activation preserves the phenotype of human pluripotent stem cells (hPSCs). CXCR2 suppression decreased proliferation of hPSCs, reduced the expression of p-mTOR (as we observed in RPPA analysis) and protein levels of some markers of stemness, and promoted differentiation of hPSCs along the mesodermal and endodermal lineage [53].

We do note some limitations of our study. This work was designed to examine the role of CXCR2 expression during the process of melanoma tumorigenesis. We clearly observed that loss of CXCR2 expression in tyrosinase-expressing cells where there was expression of mutant BRAF and loss of PTEN resulted in reduced tumor growth and lowered incidence of tumor formation. However, we did observe some chimerism in the loss of CXCR2 in the *Braf/Pten/Cxcr2*^*fl/fl*^ model. Approximately 70% of the tyrosinase expressing GFP-positive tumor cells expressed CXCR2 in the tumors arising in *Braf/Pten/CXCR2*^*WT*^ model and 30% of the tyrosinase expressing GFP + tumor cells in the *Braf/Pten/Cxcr2*^*−/−*^ model continued to express CXCR2. This chimerism has likely impacted the significance of our results, as the chimerism could be associated with more *Braf/Pten/Cxcr2*^*−/−*^ tumor formation than would have occurred if 100% of the GFP-positive tumor cells had been negative for CXCR2 expression. Moreover, in the *Braf/Pten/Cxcr2*^*WT*^ mice, we might have observed an increase in tumor growth and incidence if 100% of the tumor cells were CXCR2 positive. In the *NRas/Ink4a* model of melanoma, loss of CXCR2 expression during tumorigenesis also resulted in reduced tumor growth, without a significant reduction in tumor incidence. This discrepancy is likely due to effects of UV irradiation and the early treatment (Day1-3 post birth) with 4HT to induce NRAS mutation and loss of INK4a in this model, potentially evoking tumorigenesis mechanisms that are less dependent on CXCR2. While we did not perform staining of the *NRas/Ink4a* tumors, it is reasonable to expect some degree of chimerism in this model as well.

Our finding that loss or inhibition of Cxcr2 activity in melanocytic cells results in changes in markers associated with stemness, neural crest cells, and melanoblasts in association with a reduction of tumor formation and growth is somewhat paradoxical. However, human melanoma tumors are quite heterogeneous [48], with stem-like cell populations as well as more differentiated populations expressing MITF, TYR, and MELANA. Of note, nests of stem-like melanoma cells have been identified in metastatic lesions in head and neck cancer patients and shown to express *NANOG, OCT4, SOX2, KLF4,* and *cMYC* [54]. Moreover, melanocytes and melanoma cells have been dedifferentiated to iPSCs by transfecting in *Oct4, c-Myc,* and *Klf4* expression vectors. The resulting iPSCs express *Nanog* and *Oct4* and can be differentiated into fibroblast-like cells [55]. Our data suggest that loss of CXCR2 signaling may reduce sub-populations of melanoma cells expressing the stem cell marker *Esrrb* but increases populations with the stemness markers *Klf4, Hmga1,* and *Tfcp2l1*. Moreover, the gene expression pattern in the six functionally enriched states of tumor cells previously established by single-cell transcriptomics: melanocytic, neural crest-like, antigen-presenting, RNA processing, stem-like, and stress-like, appear to be altered with loss of Cxcr2 signaling, especially in the melanocytic state [56]. In addition to its role in maintaining stemness in a population of tumor cells, TFCP2L1 is likely also contributing to tumor suppressor in the melanoma models studied here.

## Conclusion

We demonstrate that targeted deletion of *Cxcr2* in tyrosinase-expressing melanoma precursor cells concurrent with induction of the *Braf*^*V600E*^ transgene and loss of *Pten* expression or induction of *NRas*^*Q61R*^ and loss of *Ink4a*, resulted in a significant reduction of melanoma burden. Notably, we also observed reduced expression of genes involved in growth, increased expression of genes involved in tumor suppression, and promotion of an anti-tumor immune environment when *Cxcr2* was deleted in tyrosinase-expressing melanoma precursor cells during transformation. Importantly, we show that the CXCR1/CXCR2 antagonist, SX-682, accomplishes a similar reduction in melanoma tumor burden, establishes an anti-tumor immune microenvironment, and significantly alters the transcriptional profile of melanoma cells when delivered during the transformation process. A key mechanism for these transcriptional changes involves increased expression of *Tfcp2l1*, a predicted tumor suppressive transcription factor when Cxcr2 activity is blocked.

Our data support combining CXCR1/CXCR2 antagonists with immunotherapy for melanoma patients. Consistent with this concept, we have shown that the antagonism of CXCR2 upregulates PD-L1 expression and enhances the response of melanoma cells to anti-PD-1 [9]. Moreover, CXCR1/CXCR2 antagonists combined with anti-PD-1 are currently in clinical trials for the treatment of melanoma (NCT03161431). Moving forward, it will be essential to identify the subset of patients most likely to respond to this combination therapy and to develop protocols for maximal response.

## Supplementary Information

**Additional file 1.**

## Data Availability

The datasets supporting the conclusions of this article are available in the Gene Expression Omnibus under accession GSE223290.

## Abbreviations

CXCR2: C-X-C Motif Chemokine Receptor 2
INK4a: Inhibitor of cyclin-dependent kinase 4a
PTEN: Phosphatase and tensin Homolog
TFCP2L1: Transcription Factor CP2 Like 1
MDSC: Myeloid-derived suppressor cells
TME: Tumor microenvironment
PI3K: Phosphatidylinositol-3-kinase
MAPK: Mitogen-activated protein kinase
AKT: Protein kinase B
NF-κB: Nuclear factor kappa-light-chain-enhancer of activated B cells
KC: Murine CXC chemokine likely equivalent to human CXCL1
MIP-2: Macrophage-inflammatory protein-2-Murine CXC chemokine likely equivalent to human CXCL8
LIX1: Lipopolysaccharide-induced CXC chemokine 1-Murine CXC chemokine likely equivalent to CXCL5
TCGA: The cancer genome atlas
GEPIA: Gene expression profiling interactive analysis
GSEA: Gene set enrichment analysis
WGCNA: Weighted gene co-expression network analysis
PD-1: Programmed cell death protein 1
CRE: Cis regulatory element
4-HT: Hydroxytamoxifen
mT/mG: A cell membrane-localized Tomato (mT) and EGFP (mG) as a two-color fluorescent Cre-reporter allele
RNAseq: RNA sequencing
Tmprss11e: Transmembrane serine protease 11e
Adamts18: ADAM metallopeptidase with thrombospondin type 1 motif 18
TGM3: Transglutaminase 3
GSDMc: Gasdermin C
ELF3: E74 like ETS transcription factor 3
FCNA: Ficolin A
MLK4: Myosin light chain kinase 4
FACS: Fluorescence activated cell sorting
M-CSF: Macrophage colony-stimulating factor
VEGF: Vascular endothelial growth factor
RPPA: Reverse phosphoprotein analysis
mTOR: mammalian target of rapamycin
MMP: Matrix metalloproteinase
PAX8: Paired box gene 8
STING: Stimulator of interferon genes
TRIM25: Tripartite motif containing 25
PUMA: P53 upregulated modulator of apoptosis
BclxL: B-cell lymphoma-extra large
SMAC: Second mitochondrial activator of caspases
TSC2: Tuberous sclerosis complex 2
WTAP: Wilms tumor suppressor 1 associated protein
PLK1: Polo like kinase 1
PRAS40: The proline-rich AKT substrate of 40 kDa
HMHA1: Minor histocompatibility protein HA-1
KLF: Krupple-like factor
NANOG: Nanog homeobox protein encoding gene
OCT4: Octamer-binding transcription factor 4
*SOX2*: SRY-box transcription factor 2
ESRRB: Estrogen related receptor beta
NOTCH1: Notch receptor 1
HMGA2: High mobility group AT-hook 2
FOXD3: Forkhead box D3
ANOVA: Analysis of variance
CHIPseq: Chromatin immunoprecipitation and sequencing analysis
NHEM: Normal human epidermal melanocyte
MELANA: Melanocyte antigen.
PSCs: Pluripotent stem cells
