# Supplementary material for: CXCR2 expression during melanoma tumorigenesis controls transcriptional programs that facilitate tumor growth
Source: Mol Cancer. 2023 Jun 3;22:92. doi: 10.1186/s12943-023-01789-9 (PMC10239119; doi:10.1186/s12943-023-01789-9)
Supplement: Supplementary file 1 — Additional file 1. [file 12943_2023_1789_MOESM1_ESM.pdf]

Figure S1.

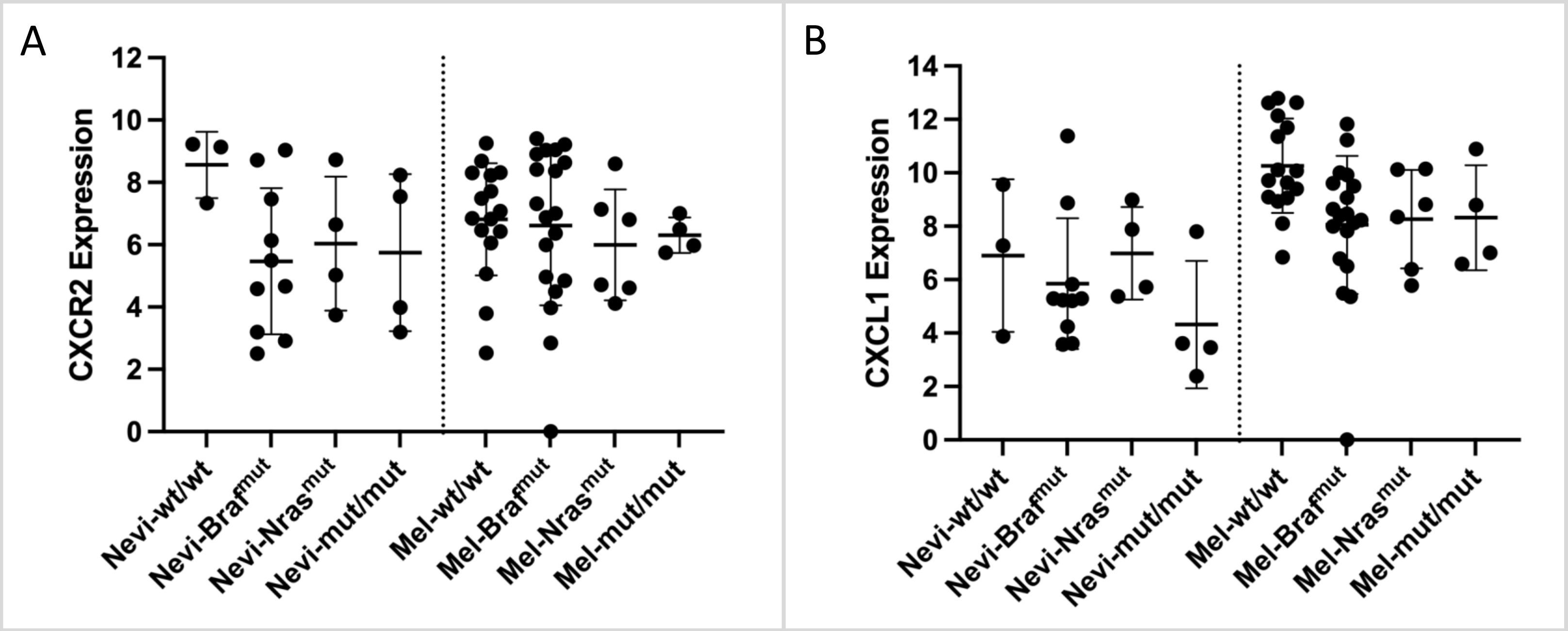
A
CXCR2 Expression
0
2
4
6
8
10
12
Nevi-wt/wt
Nevi-Braf^{mut}
Nevi-Nras^{mut}
Nevi-mut/mut
Mel-wt/wt
Mel-Braf^{mut}
Mel-Nras^{mut}
Mel-mut/mut
B
CXCL1 Expression
0
2
4
6
8
10
12
14
Nevi-wt/wt
Nevi-Braf^{mut}
Nevi-Nras^{mut}
Nevi-mut/mut
Mel-wt/wt
Mel-Braf^{mut}
Mel-Nras^{mut}
Mel-mut/mut

Figure S2.

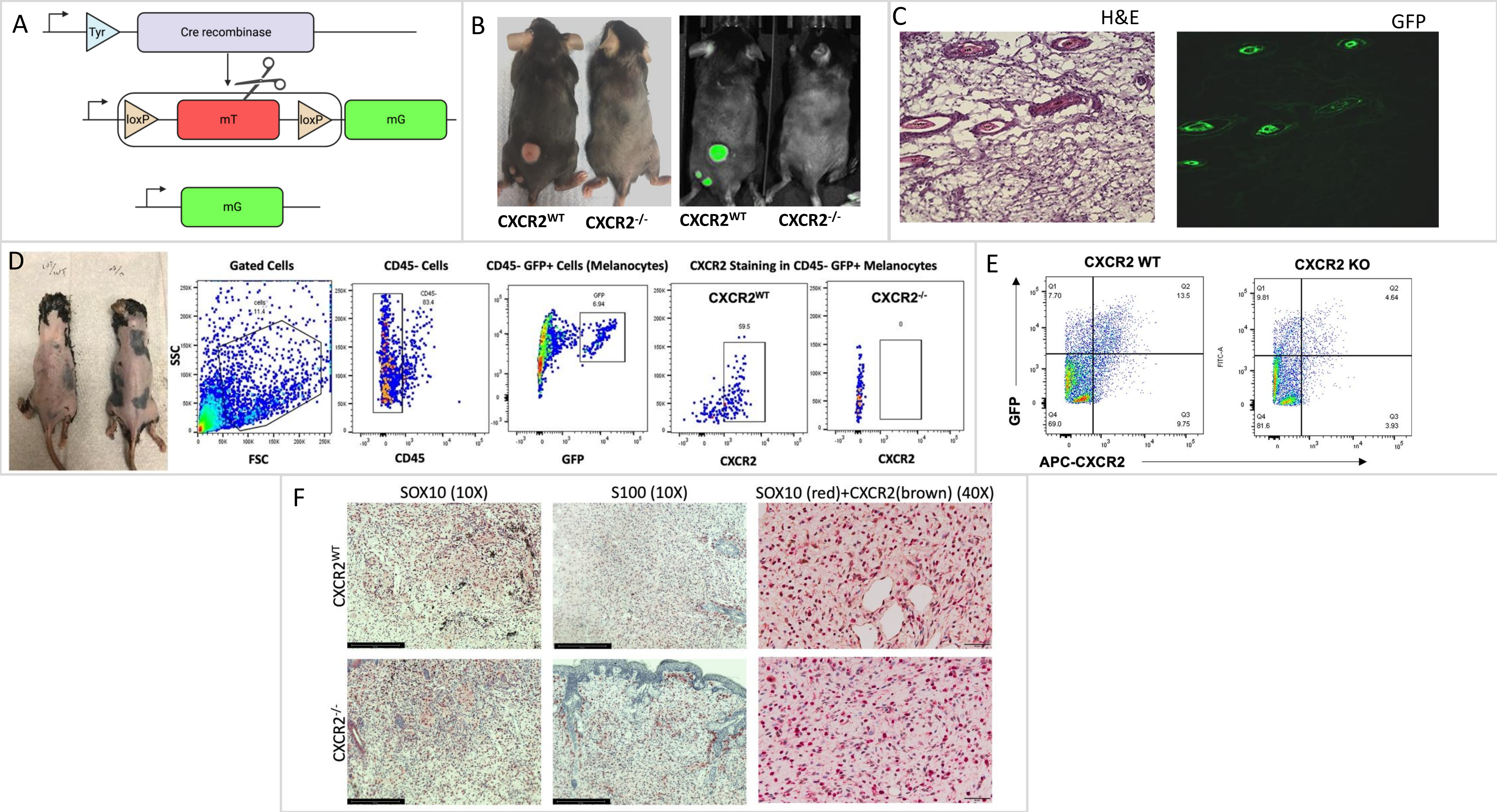
A
Tyr
Cre recombinase
loxP
mT
loxP
mG
mG
B
CXCR2WT
CXCR2-/-
CXCR2WT
CXCR2-/-
C
H&E
GFP
D
Gated Cells
CD45- Cells
CD45- GFP+ Cells (Melanocytes)
CXCR2 Staining in CD45- GFP+ Melanocytes
CXCR2WT
CXCR2-/-
SSC
FSC
CD45
GFP
CXCR2
CXCR2
E
CXCR2 WT
CXCR2 KO
GFP
APC-CXCR2
F
SOX10 (10X)
S100 (10X)
SOX10 (red)+CXCR2(brown) (40X)
CXCR2WT
CXCR2-/-

Figure S3

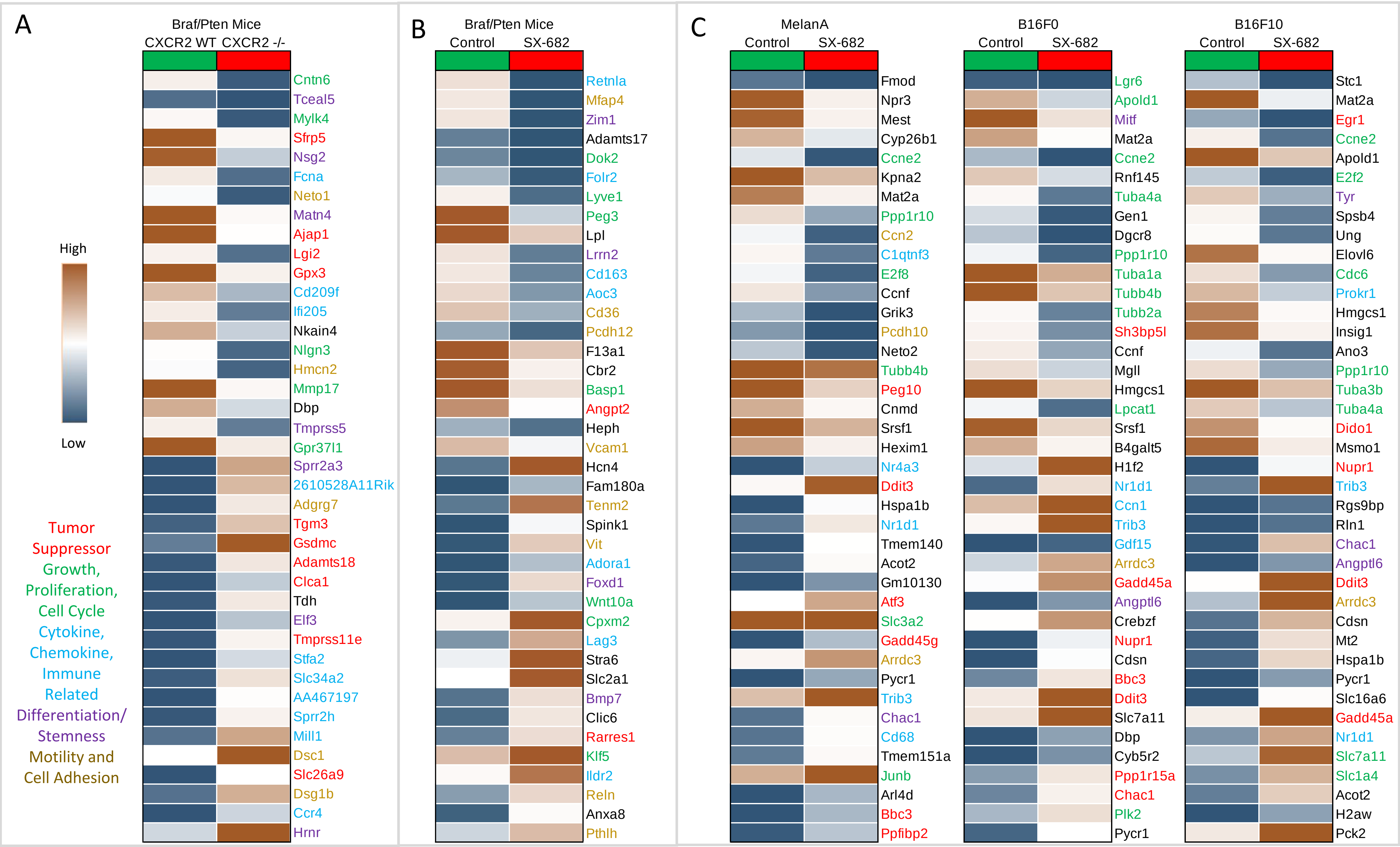
A
Braf/Pten Mice
CXCR2 WT
CXCR2 -/-
Cntn6
Tceal5
Mylk4
Sfrp5
Nsg2
Fcna
Neto1
Matn4
Ajap1
Lgi2
Gpx3
Cd209f
Ifi205
Nkain4
Nlgn3
Hmcn2
Mmp17
Dbp
Tmprss5
Gpr37l1
Sprr2a3
2610528A11Rik
Adgrg7
Tgm3
Gsdmc
Adamts18
Clca1
Tdh
Elf3
Tmprss11e
Stfa2
Slc34a2
AA467197
Sprr2h
Mill1
Dsc1
Slc26a9
Dsg1b
Ccr4
Hrnr
High
Low
Tumor Suppressor
Growth, Proliferation, Cell Cycle
Cytokine, Chemokine, Immune Related
Differentiation/ Stemness
Motility and Cell Adhesion
B
Braf/Pten Mice
Control
SX-682
Retnla
Mfap4
Zim1
Adamts17
Dok2
Folr2
Lyve1
Peg3
Lpl
Lrrn2
Cd163
Aoc3
Cd36
Pcdh12
F13a1
Cbr2
Basp1
Angpt2
Heph
Vcam1
Hcn4
Fam180a
Tenm2
Spink1
Vit
Adora1
Foxd1
Wnt10a
Cpxm2
Lag3
Stra6
Slc2a1
Bmp7
Clic6
Rarres1
Klf5
Ildr2
Reln
Anxa8
Pthlh
C
MelanA
Control
SX-682
Fmod
Npr3
Mest
Cyp26b1
Ccne2
Kpna2
Mat2a
Ppp1r10
Ccn2
C1qtnf3
E2f8
Ccnf
Grik3
Pcdh10
Neto2
Tubb4b
Peg10
Cnmd
Srsf1
Hexim1
Nr4a3
Ddit3
Hspa1b
Nr1d1
Tmem140
Acot2
Gm10130
Atf3
Slc3a2
Gadd45g
Arrdc3
Pycr1
Trib3
Chac1
Cd68
Tmem151a
Junb
Arl4d
Bbc3
Ppfibp2
B16F0
Control
SX-682
Lgr6
Apold1
Mitf
Mat2a
Ccne2
Rnf145
Tuba4a
Gen1
Dgcr8
Ppp1r10
Tuba1a
Tubb4b
Tubb2a
Sh3bp5l
Ccnf
Mgll
Hmgcs1
Lpcat1
Srsf1
B4galt5
H1f2
Nr1d1
Ccn1
Trib3
Gdf15
Arrdc3
Gadd45a
Angptl6
Crebzf
Nupr1
Cdsn
Bbc3
Ddit3
Slc7a11
Dbp
Cyb5r2
Ppp1r15a
Chac1
Plk2
Pycr1
B16F10
Control
SX-682
Stc1
Mat2a
Egr1
Ccne2
Apold1
E2f2
Tyr
Spsb4
Ung
Elovl6
Cdc6
Prokr1
Hmgcs1
Insig1
Ano3
Ppp1r10
Tuba3b
Tuba4a
Dido1
Msmo1
Nupr1
Trib3
Rgs9bp
Rln1
Chac1
Angptl6
Ddit3
Arrdc3
Cdsn
Mt2
Hspa1b
Pycr1
Slc16a6
Gadd45a
Nr1d1
Slc7a11
Slc1a4
Acot2
H2aw
Pck2

Figure S4.

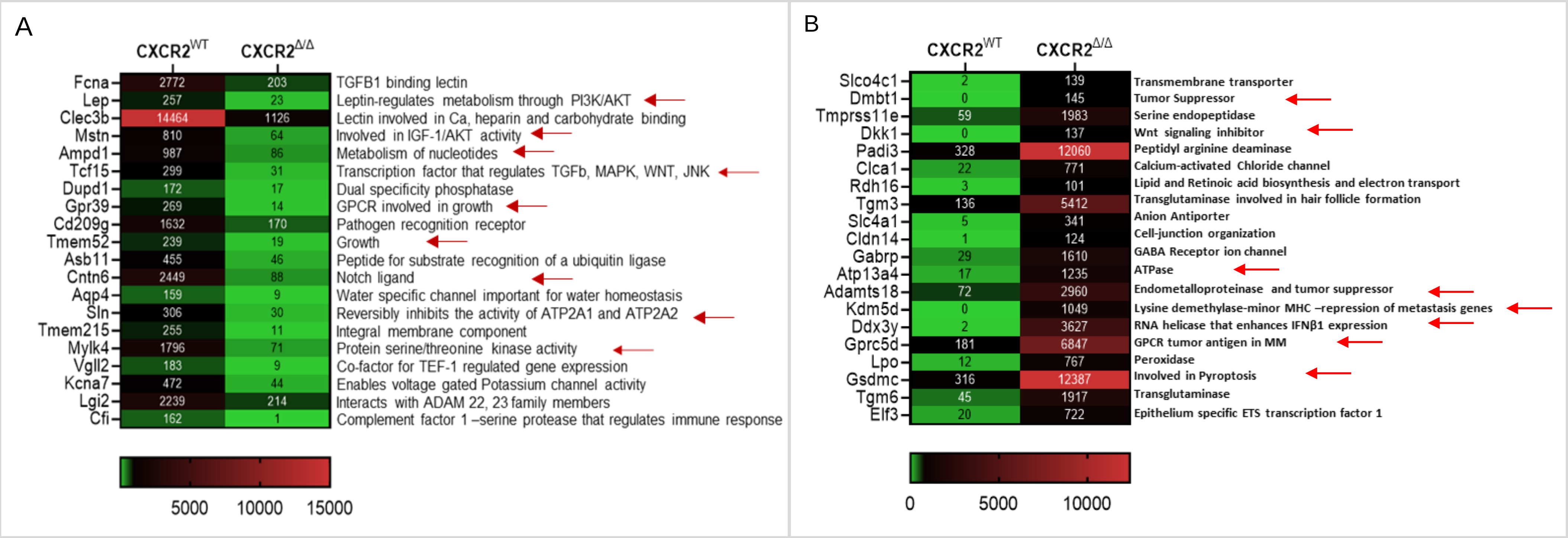
A
CXCR2WT
CXCR2Δ/Δ
Fcna 2772 203 TGFB1 binding lectin
Lep 257 23 Leptin-regulates metabolism through PI3K/AKT
Clec3b 14464 1126 Lectin involved in Ca, heparin and carbohydrate binding
Mstn 810 64 Involved in IGF-1/AKT activity
Ampd1 987 86 Metabolism of nucleotides
Tcf15 299 31 Transcription factor that regulates TGFb, MAPK, WNT, JNK
Dupd1 172 17 Dual specificity phosphatase
Gpr39 269 14 GPCR involved in growth
Cd209g 1632 170 Pathogen recognition receptor
Tmem52 239 19 Growth
Asb11 455 46 Peptide for substrate recognition of a ubiquitin ligase
Cntn6 2449 88 Notch ligand
Aqp4 159 9 Water specific channel important for water homeostasis
Sln 306 30 Reversibly inhibits the activity of ATP2A1 and ATP2A2
Tmem215 255 11 Integral membrane component
Mylk4 1796 71 Protein serine/threonine kinase activity
Vgll2 183 9 Co-factor for TEF-1 regulated gene expression
Kcna7 472 44 Enables voltage gated Potassium channel activity
Lgi2 2239 214 Interacts with ADAM 22, 23 family members
Cfi 162 1 Complement factor 1 –serine protease that regulates immune response
5000 10000 15000
B
CXCR2WT
CXCR2Δ/Δ
Slco4c1 2 139 Transmembrane transporter
Dmbt1 0 145 Tumor Suppressor
Tmprss11e 59 1983 Serine endopeptidase
Dkk1 0 137 Wnt signaling inhibitor
Padi3 328 12060 Peptidyl arginine deaminase
Clca1 22 771 Calcium-activated Chloride channel
Rdh16 3 101 Lipid and Retinoic acid biosynthesis and electron transport
Tgm3 136 5412 Transglutaminase involved in hair follicle formation
Slc4a1 5 341 Anion Antiporter
Cldn14 1 124 Cell-junction organization
Gabrp 29 1610 GABA Receptor ion channel
Atp13a4 17 1235 ATPase
Adamts18 72 2960 Endometalloproteinase and tumor suppressor
Kdm5d 0 1049 Lysine demethylase-minor MHC –repression of metastasis genes
Ddx3y 2 3627 RNA helicase that enhances IFNβ1 expression
Gprc5d 181 6847 GPCR tumor antigen in MM
Lpo 12 767 Peroxidase
Gsdmc 316 12387 Involved in Pyroptosis
Tgm6 45 1917 Transglutaminase
Elf3 20 722 Epithelium specific ETS transcription factor 1
0 5000 10000

Figure S5.

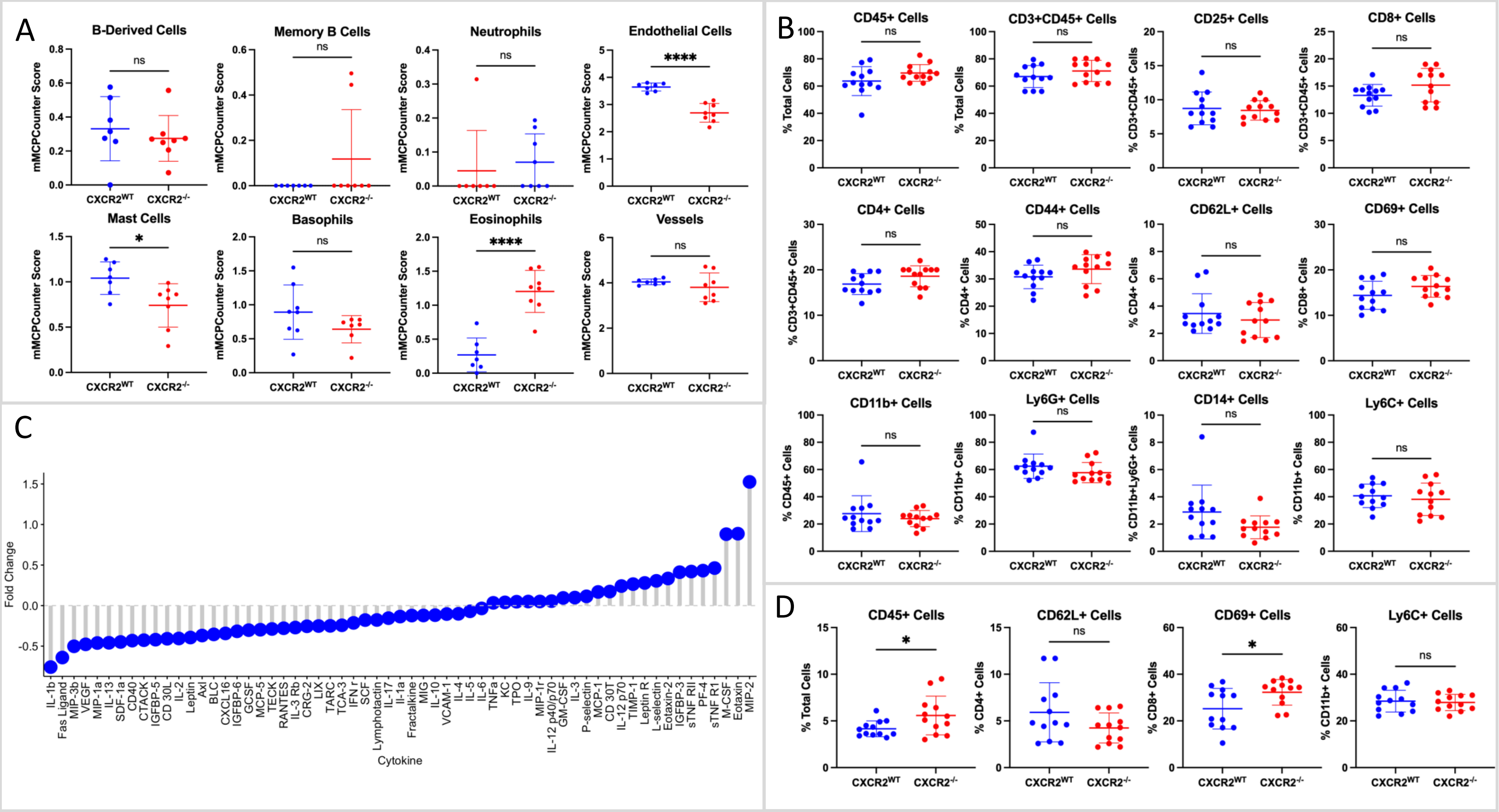
A
B-Derived Cells
Memory B Cells
Neutrophils
Endothelial Cells
Mast Cells
Basophils
Eosinophils
Vessels
mMCPCounter Score
CXCR2WT
CXCR2-/-
ns
*
****
B
CD45+ Cells
CD3+CD45+ Cells
CD25+ Cells
CD8+ Cells
CD4+ Cells
CD44+ Cells
CD62L+ Cells
CD69+ Cells
CD11b+ Cells
Ly6G+ Cells
CD14+ Cells
Ly6C+ Cells
% Total Cells
% CD3+CD45+ Cells
% CD4+ Cells
% CD8+ Cells
% CD45+ Cells
% CD11b+ Cells
% CD11b+Ly6G+ Cells
C
Fold Change
Cytokine
IL-1b
Fas Ligand
MIP-3b
VEGF
MIP-1a
IL-13
SDF-1a
CD40
CTACK
IGFBP-5
CD 30L
IL-2
Leptin
Axl
BLC
CXCL16
IGFBP-6
GCSF
MCP-5
TECK
RANTES
IL-3 Rb
CRG-2
LIX
TARC
TCA-3
IFN r
SCF
Lymphotactin
IL-17
Il-1a
Fractalkine
MIG
IL-10
VCAM-1
IL-4
IL-5
IL-6
TNFa
KC
TPO
IL-9
MIP-1r
IL-12 p40/p70
GM-CSF
IL-3
P-selectin
MCP-1
CD 30T
IL-12 p70
TIMP-1
Leptin R
L-selectin
Eotaxin-2
IGFBP-3
sTNF RII
PF-4
sTNF R1
M-CSF
Eotaxin
MIP-2
D
CD45+ Cells
CD62L+ Cells
CD69+ Cells
Ly6C+ Cells

Figure S6.

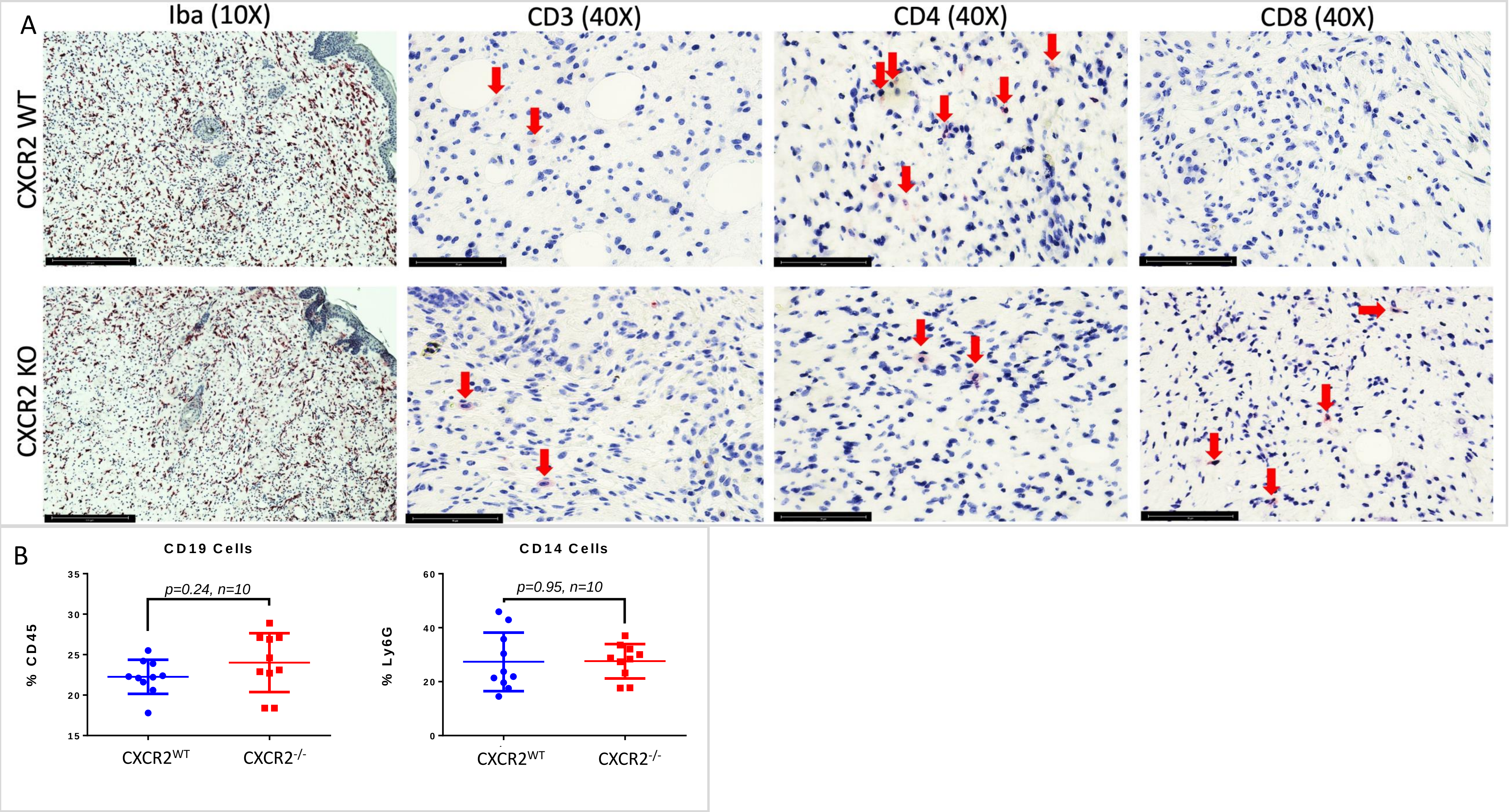
A
Iba (10X)
CD3 (40X)
CD4 (40X)
CD8 (40X)
CXCR2 WT
CXCR2 KO
B
CD19 Cells
p=0.24, n=10
% CD45
35
30
25
20
15
CXCR2WT
CXCR2-/-
CD14 Cells
p=0.95, n=10
% Ly6G
60
40
20
0
CXCR2WT
CXCR2-/-

Figure S7.

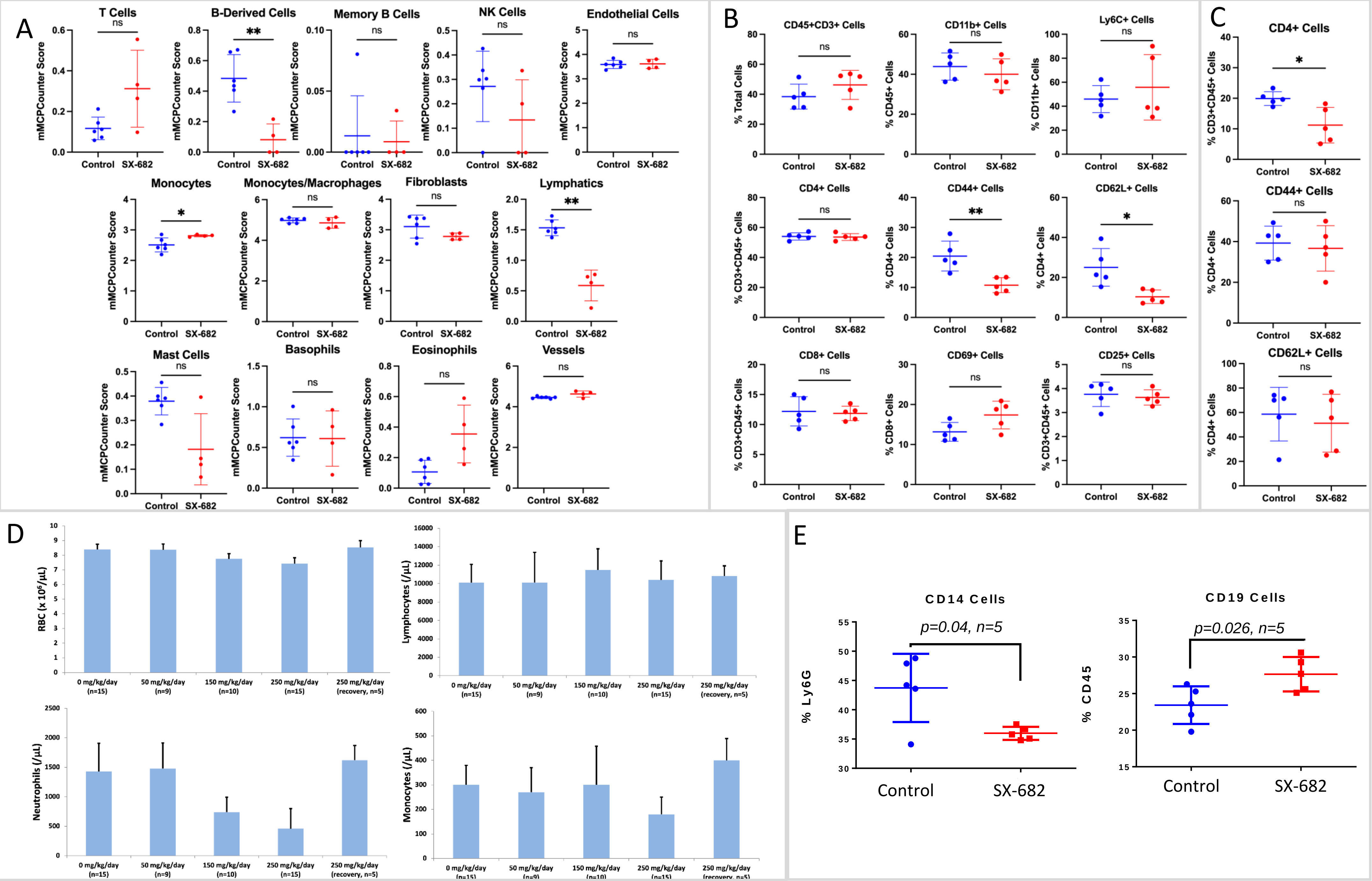

A
T Cells
B-Derived Cells
Memory B Cells
NK Cells
Endothelial Cells
Monocytes
Monocytes/Macrophages
Fibroblasts
Lymphatics
Mast Cells
Basophils
Eosinophils
Vessels
mMCPCounter Score
Control
SX-682
ns
*
**
B
CD45+CD3+ Cells
CD11b+ Cells
Ly6C+ Cells
CD4+ Cells
CD44+ Cells
CD62L+ Cells
CD8+ Cells
CD69+ Cells
CD25+ Cells
% Total Cells
% CD45+ Cells
% CD11b+ Cells
% CD3+CD45+ Cells
% CD4+ Cells
% CD8+ Cells
C
CD4+ Cells
CD44+ Cells
CD62L+ Cells
D
RBC (x 10^6/μL)
Lymphocytes (/μL)
Neutrophils (/μL)
Monocytes (/μL)
0 mg/kg/day (n=15)
50 mg/kg/day (n=9)
150 mg/kg/day (n=10)
250 mg/kg/day (n=15)
250 mg/kg/day (recovery, n=5)
E
CD14 Cells
CD19 Cells
p=0.04, n=5
p=0.026, n=5
% Ly6G
% CD45
Control
SX-682

Figure S8.

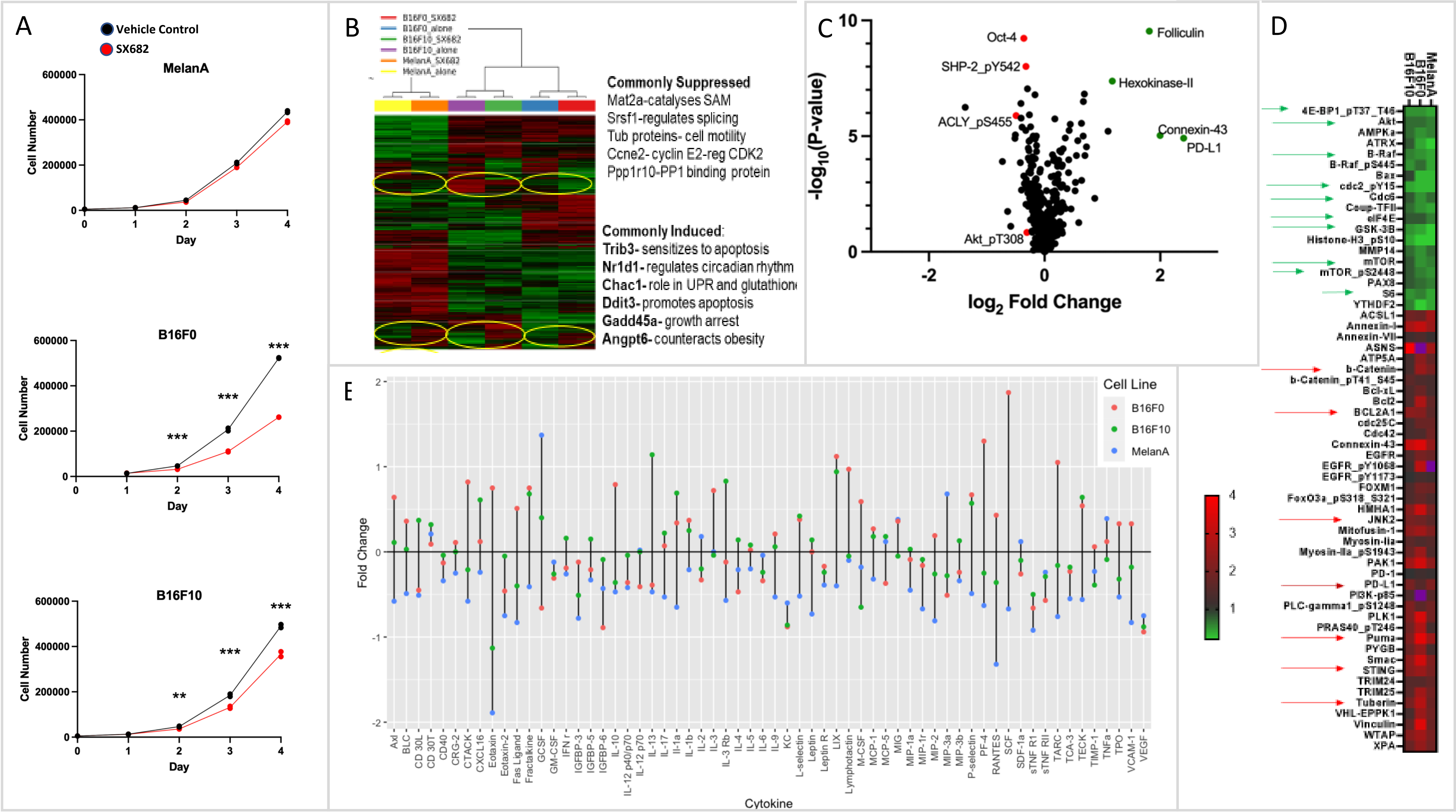
A
Vehicle Control
SX682
MelanA
B16F0
B16F10
Cell Number
Day
B
Commonly Suppressed
Mat2a-catalyses SAM
Srsf1-regulates splicing
Tub proteins- cell motility
Ccne2- cyclin E2-reg CDK2
Ppp1r10-PP1 binding protein
Commonly Induced:
Trib3- sensitizes to apoptosis
Nr1d1- regulates circadian rhythm
Chac1- role in UPR and glutathion
Ddit3- promotes apoptosis
Gadd45a- growth arrest
Angpt6- counteracts obesity
C
Oct-4
SHP-2_pY542
ACLY_pS455
Akt_pT308
Folliculin
Hexokinase-II
Connexin-43
PD-L1
-log10(P-value)
log2 Fold Change
D
E
Cell Line
B16F0
B16F10
MelanA
Fold Change
Cytokine

Figure S9.

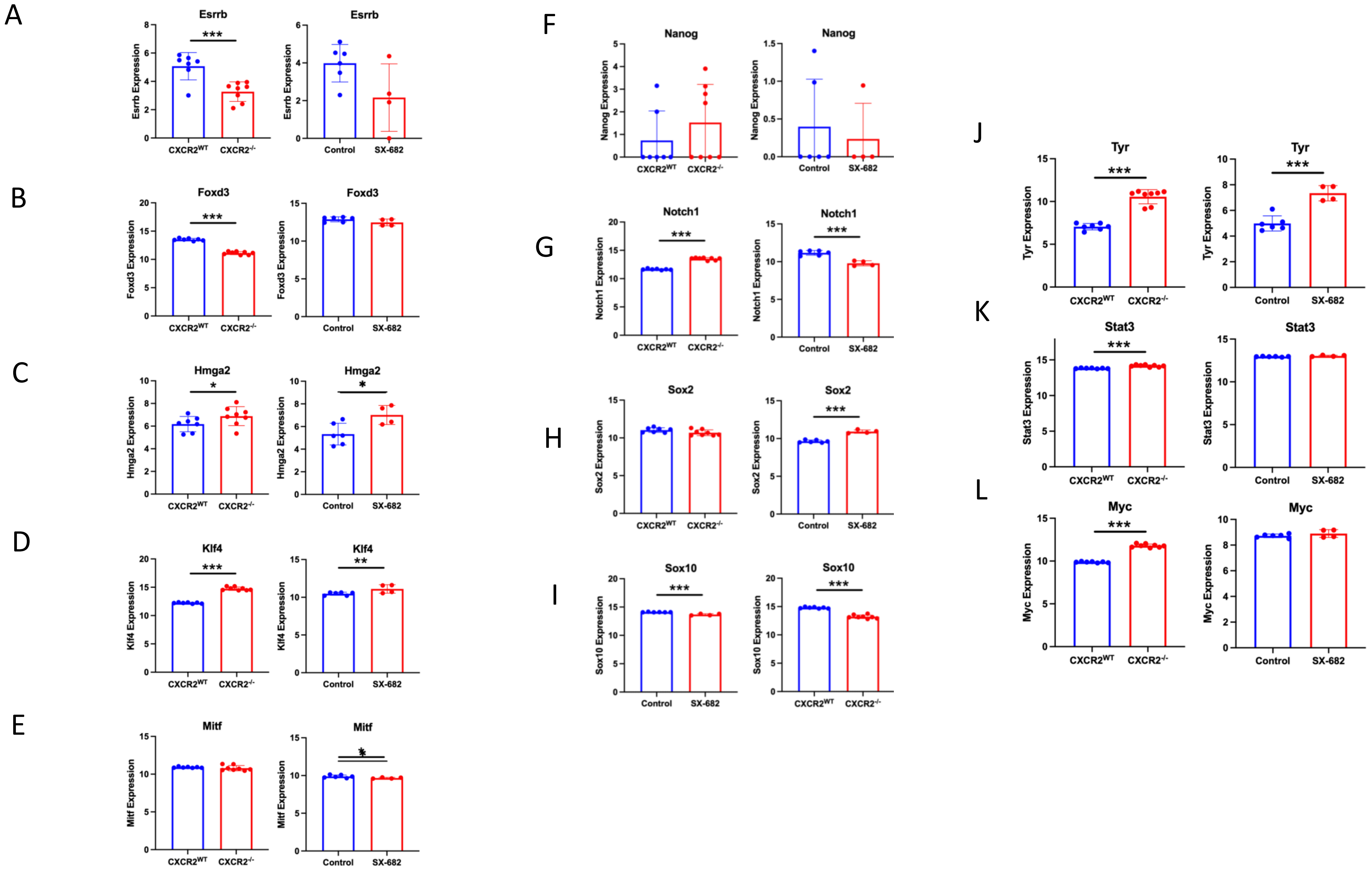

A
Esrrb
Esrrb Expression
CXCR2WT
CXCR2-/-
Control
SX-682
B
Foxd3
Foxd3 Expression
C
Hmga2
Hmga2 Expression
D
Klf4
Klf4 Expression
E
Mitf
Mitf Expression
F
Nanog
Nanog Expression
G
Notch1
Notch1 Expression
H
Sox2
Sox2 Expression
I
Sox10
Sox10 Expression
J
Tyr
Tyr Expression
K
Stat3
Stat3 Expression
L
Myc
Myc Expression

Figure S10

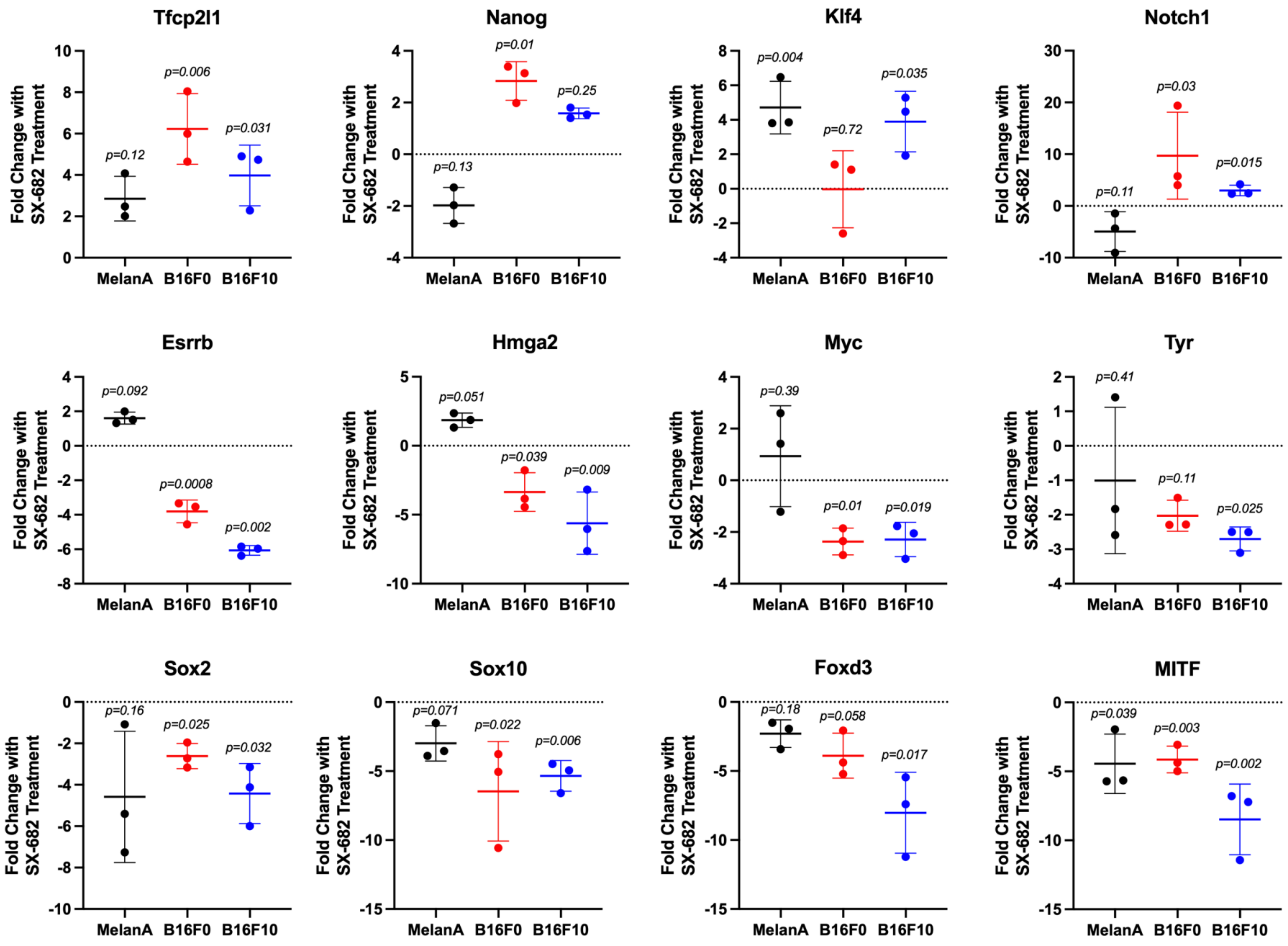
Tfcp2l1
p=0.12
p=0.006
p=0.031
Nanog
p=0.13
p=0.01
p=0.25
Klf4
p=0.004
p=0.72
p=0.035
Notch1
p=0.11
p=0.03
p=0.015
Esrrb
p=0.092
p=0.0008
p=0.002
Hmga2
p=0.051
p=0.039
p=0.009
Myc
p=0.39
p=0.01
p=0.019
Tyr
p=0.41
p=0.11
p=0.025
Sox2
p=0.16
p=0.025
p=0.032
Sox10
p=0.071
p=0.022
p=0.006
Foxd3
p=0.18
p=0.058
p=0.017
MITF
p=0.039
p=0.003
p=0.002
Fold Change with SX-682 Treatment
MelanA
B16F0
B16F10

Figure S11

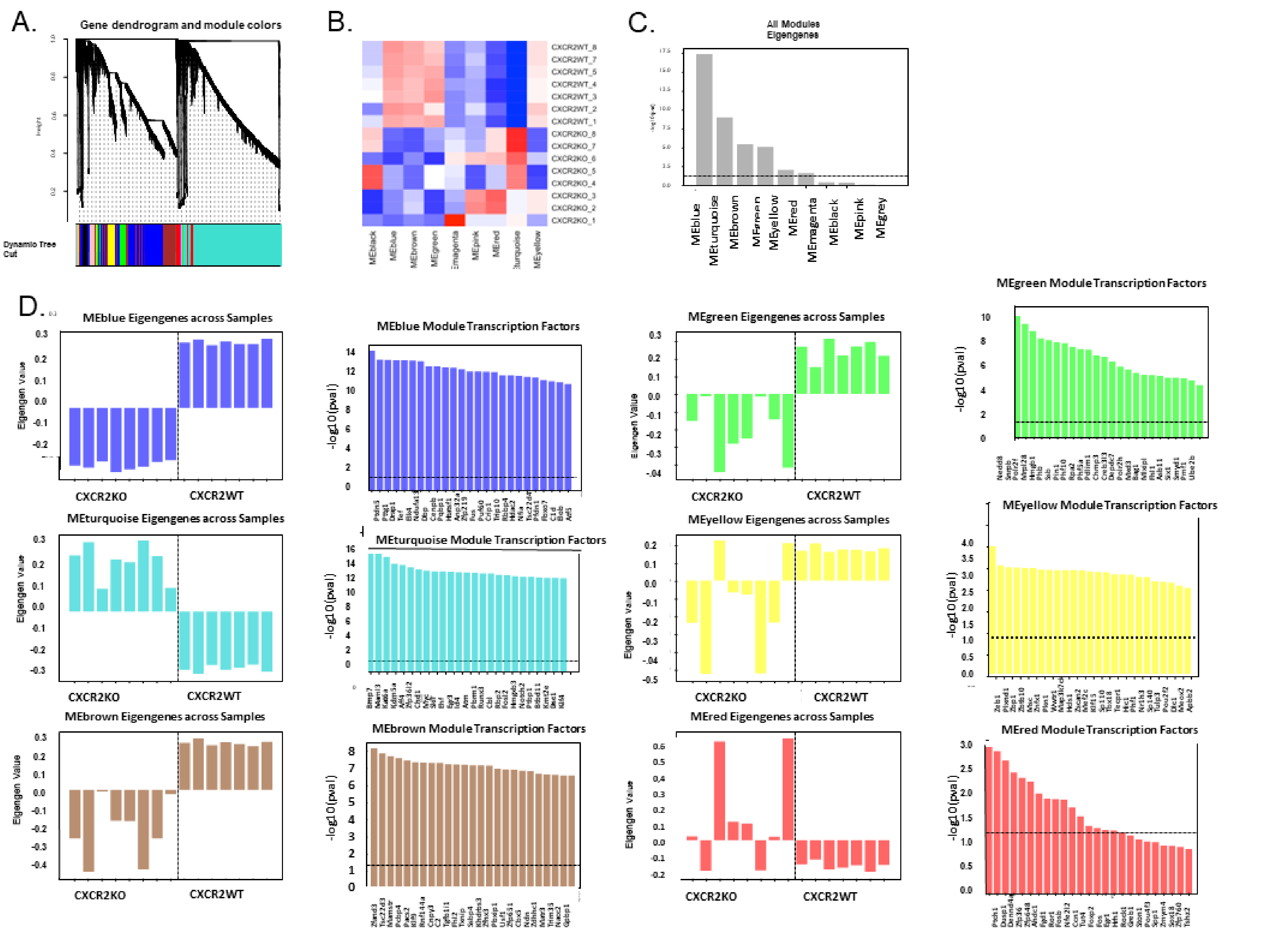

A.
Gene dendrogram and module colors
Dynamic Tree Cut
B.
CXCR2WT_8
CXCR2WT_7
CXCR2WT_5
CXCR2WT_4
CXCR2WT_3
CXCR2WT_2
CXCR2WT_1
CXCR2KO_8
CXCR2KO_7
CXCR2KO_6
CXCR2KO_5
CXCR2KO_4
CXCR2KO_3
CXCR2KO_2
CXCR2KO_1
MEblack
MEblue
MEbrown
MEgreen
MEpink
MEred
MEyellow
C.
All Modules Eigengenes
MEblue
MEturquoise
MEbrown
MEgreen
MEyellow
MEred
MEmagenta
MEblack
MEpink
MEgrey
D.
MEblue Eigengenes across Samples
Eigengen Value
CXCR2KO
CXCR2WT
MEblue Module Transcription Factors
-log10(pval)
MEgreen Eigengenes across Samples
MEgreen Module Transcription Factors
MEturquoise Eigengenes across Samples
MEturquoise Module Transcription Factors
MEyellow Eigengenes across Samples
MEyellow Module Transcription Factors
MEbrown Eigengenes across Samples
MEbrown Module Transcription Factors
MEred Eigengenes across Samples
MEred Module Transcription Factors

Figure S12

A. Terms from Genes with Increased Expression

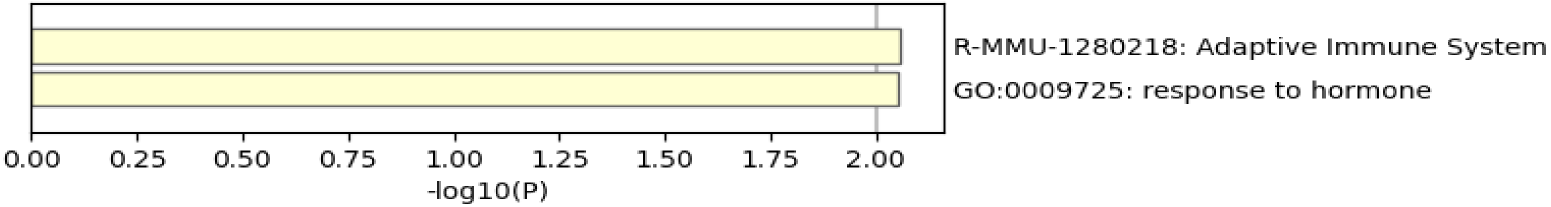

B. Terms from Genes with Decreased Expression

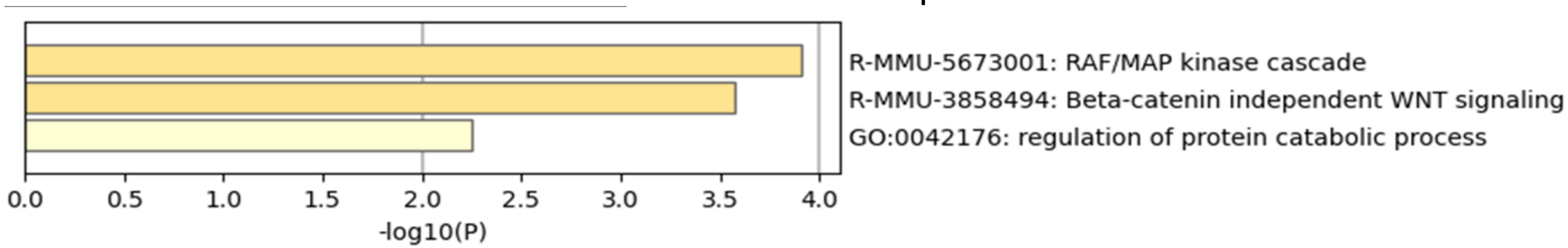

.C. Gene Overlap Analysis Heat Map of Selected GO Parent Files

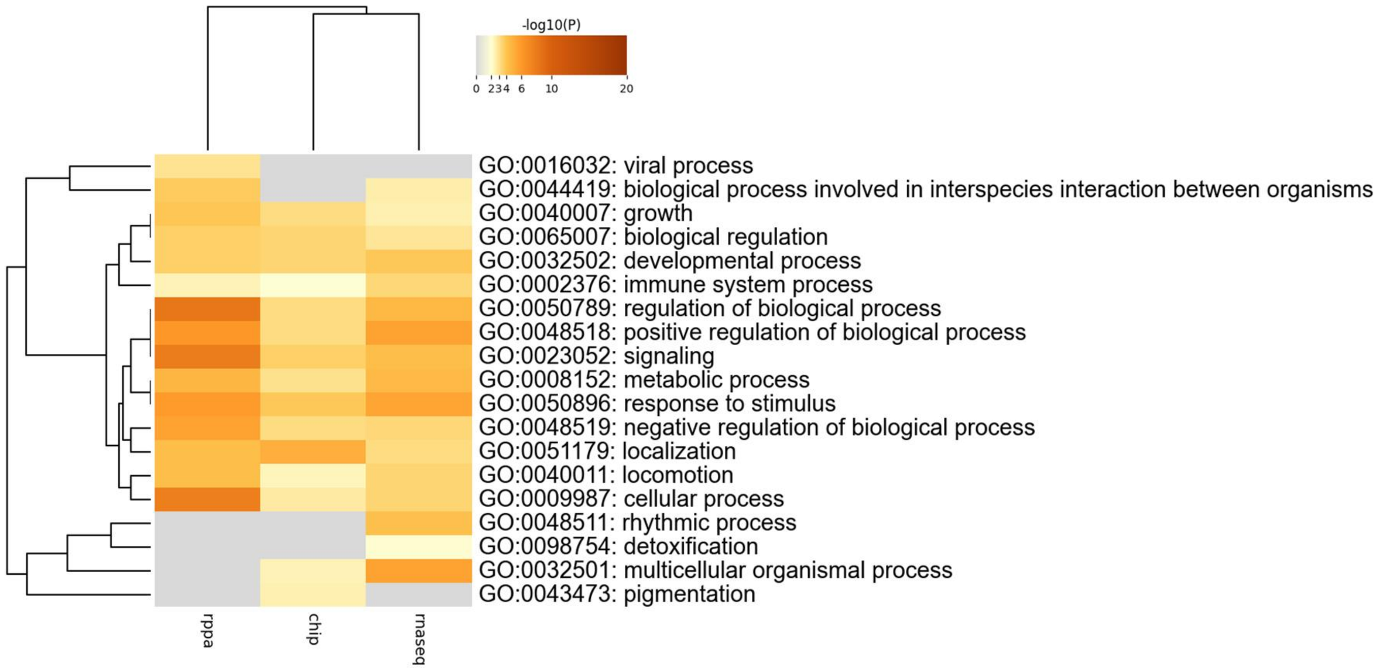

Gene Overlap Analysis—Expanded via Shared Enriched Ontologies

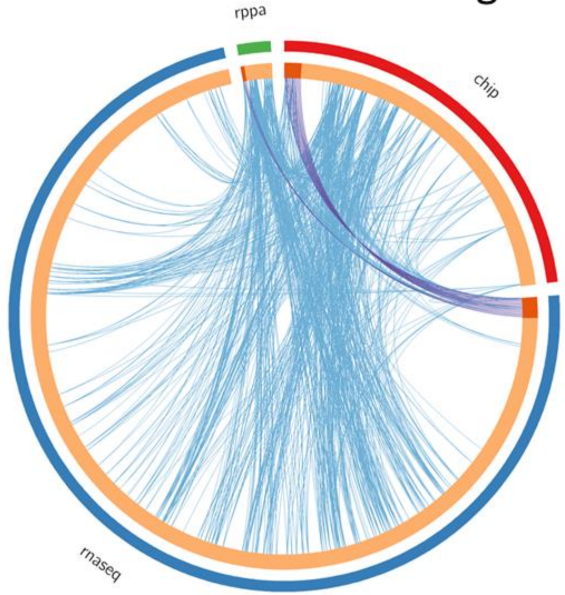

Figure S12D

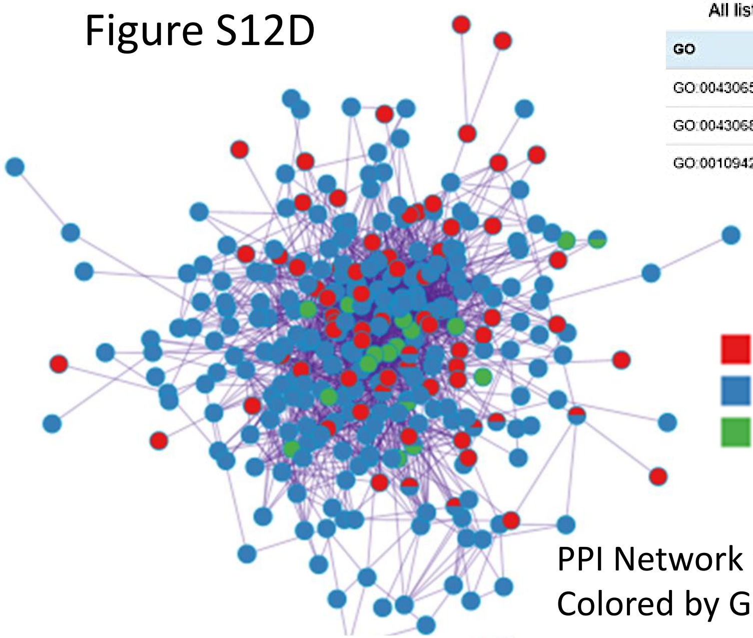

PPI Network for Lists Merged Colored by Gene Origin in Studies

All lists merged Colored by Cluster(Full Connection)

GO	Description	Log10(P)
GO:0043065	positive regulation of apoptotic process	-21.9
GO:0043068	positive regulation of programmed cell death	-21.6
GO:0010942	positive regulation of cell death	-19.5

Figure S12E

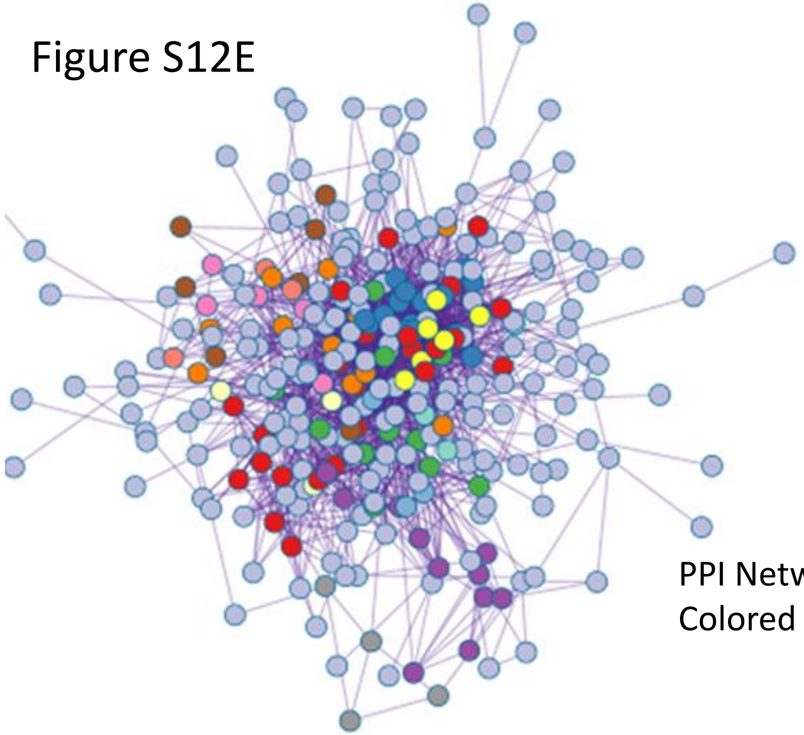

PPI Network for Lists Merged Colored by MCODE ID

All lists merged Colored by Cluster(Keep MCODE Nodes Only)

Color	MCODE	GO	Description	Log10(P)
	MCODE_1	R-MMU-212300	PRC2 methylates histones and DNA	-15.6
	MCODE_1	R-MMU-8936459	RUNX1 regulates genes involved in megakaryocyte differentiation and platelet function	-15.4
	MCODE_1	R-MMU-212165	Epigenetic regulation of gene expression	-14.3
	MCODE_2	WP6	Integrin-mediated cell adhesion	-17.3
	MCODE_2	WP85	Focal adhesion	-17.1
	MCODE_2	mmu04510	Focal adhesion - Mus musculus (house mouse)	-14.5
	MCODE_3	GO:0006366	transcription by RNA polymerase II	-6.4
	MCODE_3	GO:0006351	DNA-templated transcription	-5.6
	MCODE_3	GO:0097659	nucleic acid-templated transcription	-5.6
	MCODE_4	mmu00480	Glutathione metabolism - Mus musculus (house mouse)	-12.2
	MCODE_4	GO:0006749	glutathione metabolic process	-11.9
	MCODE_4	R-MMU-156590	Glutathione conjugation	-11.2
	MCODE_5	R-MMU-5358351	Signaling by Hedgehog	-8.3
	MCODE_5	R-MMU-2262752	Cellular responses to stress	-7.4
	MCODE_5	R-MMU-8953897	Cellular responses to stimuli	-7.4
	MCODE_6	GO:0007169	transmembrane receptor protein tyrosine kinase signaling pathway	-7.3
	MCODE_6	GO:0030335	positive regulation of cell migration	-6.5
	MCODE_6	GO:2000147	positive regulation of cell motility	-6.4
	MCODE_7	mmu04710	Circadian rhythm - Mus musculus (house mouse)	-9.7
	MCODE_7	GO:0032922	circadian regulation of gene expression	-8.3
	MCODE_7	GO:0007623	circadian rhythm	-7.2
	MCODE_8	R-MMU-72187	mRNA 3'-end processing	-9.7
	MCODE_8	R-MMU-73856	RNA Polymerase II Transcription Termination	-9.4
	MCODE_8	GO:0006397	mRNA processing	-8.5
	MCODE_9	mmu00900	Terpenoid backbone biosynthesis - Mus musculus (house mouse)	-11.9
	MCODE_9	WP4346	Cholesterol metabolism with Bloch and Kandutsch-Russell pathways	-10.5
	MCODE_9	mmu00650	Butanoate metabolism - Mus musculus (house mouse)	-8.1
	MCODE_10	GO:1900745	positive regulation of p38MAPK cascade	-8.6
	MCODE_10	mmu05216	Thyroid cancer - Mus musculus (house mouse)	-8.3
	MCODE_10	GO:1900744	regulation of p38MAPK cascade	-8.1
	MCODE_13	R-MMU-1280218	Adaptive Immune System	-4.5
	MCODE_14	GO:0070227	lymphocyte apoptotic process	-7.7
	MCODE_14	GO:0071887	leukocyte apoptotic process	-7.5
	MCODE_14	GO:0008630	intrinsic apoptotic signaling pathway in response to DNA damage	-7.2

Figure S13

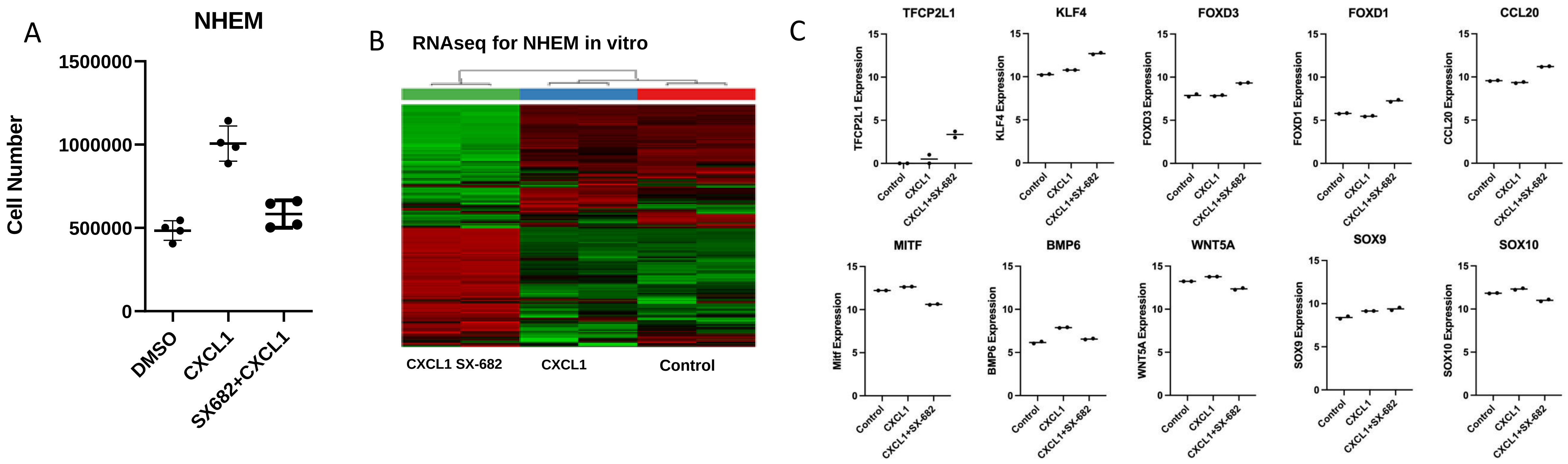

A
NHEM
Cell Number
1500000
1000000
500000
0
DMSO
CXCL1
SX682+CXCL1
B
RNAseq for NHEM in vitro
CXCL1 SX-682
CXCL1
Control
C
TFCP2L1
KLF4
FOXD3
FOXD1
CCL20
MITF
BMP6
WNT5A
SOX9
SOX10
TFCP2L1 Expression
KLF4 Expression
FOXD3 Expression
FOXD1 Expression
CCL20 Expression
Mitf Expression
BMP6 Expression
WNT5A Expression
SOX9 Expression
SOX10 Expression
Control
CXCL1
CXCL1+SX-682

Table S1

Cytokines, Chemokines, and Interleukins		
	CXCL1 vs. Control	CXCL1 + SX-682 vs. CXCL1 alone
Gene	log2FC (adj. p value)	log2FC (adj. p value)
CCL18	-0.388 (0.555)	-1.766 (9.67e-4)
CCL2	-0.426 (1.19e-22)	-6.537 (<1e-310)
CCL20	-0.298 (0.044)	1.612 (1.42e-68)
CCL7	-0.405 (0.052)	-9.397 (2.18e-10)
CCL8	0.009 (0.982)	-9.753 (4e-11)
CXCL1	-0.345 (2.9e-19)	-4.033 (<1e-310)
CXCL10	-1.476 (4.06e-9)	-1.471 (1.74e-4)
CXCL11	-1.425 (2.3e-5)	-0.482 (0.324)
CXCL12	0.710 (3.74e-18)	-6.13 (1.05e-232)
CXCL2	-0.002 (0.993)	-1.219 (2.24e-46)
CXCL3	-0.361 (0.001)	-1.384 (2.45e-28)
CXCL5	-0.62 (3.86e-36)	-1.604 (6.37e-181)
CXCL6	-0.386 (5.38e-15)	-5.928 (<1e-310)
CXCL8	-0.497 (7.99e-30)	-1.319 (5.89e-199)
IL11	1.001 (2.75e-23)	-0.285 (0.002)
IL11RA	0.011 (0.97)	-1.622 (2.51e-21)
IL12RB2	0.51 (0.041)	-1.288 (2.57e-9)
IL13RA1	-0.086 (0.233)	-0.358 (1.42e-12)
IL15RA	-0.37 (0.406)	1.693 (1.46e-14)
IL16	-0.267 (0.40)	-2.293 (1.01e-14)
IL17RB	0.019 (0.971)	0.739 (5.66e-4)
IL17RD	-0.190 (0.362)	-0.465 (0.003)
IL17RE	0.229 (0.718)	-0.794 (0.042)
IL18BP	0.052 (0.907)	0.704 (3e-4)
IL18R1	0.884 (0.025)	2.688 (4.89e-60)
IL1R1	0.293 (6.71e-11)	-2.017 (<1e-310)
IL1RL1	1.968 (5.41e-5)	3.916 (6.28e-141)
IL24	-0.123 (0.204)	-0.313 (1.38e-5)
IL27RA	-0.223 (0.316)	-0.601 (4.75e-4)
IL33	0.689 (0.037)	-4.154 (1.45e-21)
IL34	-1.609 (7.45e-13)	-0.166 (0.639)
IL4R	0.027 (0.791)	-0.494 (6.65e-22)
IL6	0.025 (0.771)	-0.82 (6.57e-77)
IL6R	0.089 (0.59)	-1.611 (4.21e-51)
IL7	-0.569 (0.023)	1.122 (3.5e-10)
IL7R	-0.259 (0.487)	-1.025 (4.34e-4)

Table S2

TNF Related Cytokines and Interferons		
	CXCL1 vs. Control	CXCL1 + SX-682 vs. CXCL1 alone
Gene	log2FC (adj. p value)	log2FC (adj. p value)
C1QTNF1	-0.135 (0.008)	-1.446 (6.63e-243)
C1QTNF2	-1.013 (0.017)	-4.923 (7.33e-8)
C1QTNF6	0.142 (0.246)	-2.543 (4.08e-117)
TNFAIP1	0.011 (0.921)	-0.22 (2.66e-5)
TNFAIP2	-0.345 (0.004)	-1.283 (4.44e-24)
TNFAIP3	-0.261 (5.54e-6)	-1.066 (1.15e-79)
TNFAIP6	0.044 (0.873)	-2.854 (6.99e-50)
TNFAIP8	-0.707 (4.81e-18)	-1.323 (2.97e-38)
TNFAIP8L1	-0.334 (0.044)	-2.102 (1.8e-33)
TNFAIP8L3	-0.048 (0.696)	-0.894 (3.21e-36)
TNFRSF10A	0.051 (0.822)	0.307 (0.006)
TNFRSF10B	0.012 (0.899)	1.231 (4.85e-177)
TNFRSF10D	0.223 (0.001)	-1.663 (2.06e-136)
TNFRSF11B	-0.168 (0.235)	-2.937 (8.15e-91)
TNFRSF12A	-0.194 (0.428)	0.659 (8.34e-6)
TNFRSF14	0.245 (0.001)	0.047 (0.515)
TNFRSF18	-0.069 (0.942)	-2.208 (4.24e-4)
TNFRSF19	0.093 (0.344)	-2.891 (3.17e-230)
TNFRSF1A	0.294 (2.96e-11)	-0.989 (6.79e-127)
TNFRSF1B	-0.151 (0.0507)	-1.757 (3.73e-131)
TNFRSF21	0.094 (0.10)	-3.004 (<1e-310)
TNFSF11	0.898 (1.46e-55)	-3.465 (<1e-310)
TNFSF12	-0.315 (0.031)	-1.333 (2.23e-20)
TNFSF13B	0.291 (0.145)	-3.421 (1.04e--53)
TNFSF4	-0.493 (0.052)	1.161 (2.51e-11)
IFNAR1	-0.074 (0.346)	-0.785 (7.76e-49)
IFNAR2	0.132 (0.801)	-1.343 (2.31e-5)
IFNGR1	0.144 (0.026)	0.059 (0.309)
IFNGR2	-0.109 (0.193)	-0.612 (1.45e-22)
IFNLR1	-0.022 (0.963)	-1.212 (4.26e-6)

Table S3.

Cancer	Mouse tumor model	CXCR1/2 inhibitor	Single agent activity*	Initiation of SX-682 Tx (days)**	Combination activity*, added agent(s)	Reference
**Prostate**	G: CPPSML	SX-682	Yes	3 weeks	Yes, ICB1	Nature 2017 [1]
**Melanoma**	S: B16	SX-682	Yes	10 – 14	Yes, anti-PD1	Unpublished 2016
**Melanoma**	S: Rich1.1	SX-682	Yes	-7	Yes, anti-PD1	Cancer Immunol Res 2020 [2]
**H&N, Lung**	S: MOC1, LLC	SX-682	No	10	Yes, anti-PD1, ET	JCI Insight 2019 [3]
**Lung**	G: $Pten^{fl/fl}$; $Lkb1^{fl/fl}$	SX-682	No	30 weeks	Yes, anti-PD1	JCI Insight 2019 [4]
**Colon**	S: MC38K	SX-682	Yes	7	Yes, anti-PD1	Cancer Cell 2020 [5]
**H&N**	S: MOC2	SX-682	Yes	7	Yes, KIL	Clin Cancer Res 2020 [6]
**Breast**	X: MDA-MB-231, S: 4T1	SX-682	Yes	7	Yes, bintrafusp	J Immunother Cancer 2020 [7]
**Colon**	S: MC38-CEA	SX-682	NT	7	Yes, bintra, vacc	Cancers 2021 [8]
**Breast (DCIS)**	G: caErbB2 lentivirus	SX-682	Yes	7	NT	Nat Commun 2021 [9]
**Lung**	S: $Kras^{G12D}$;$Trp53^{-/-}$	SX-682	Yes	20 weeks	Yes, SHP099	Cancer Discov 2022 [10]
**Pancreatic**	S: iKRAS, G: iKRAS	SX-682	Yes	>10	Yes, ICB2	Nature Cancer 2022 [11]

Supplemental Figure Legends:

Figure S1. Breakdown of CXCR2 (A) and CXCL1 (B) expression in nevi and melanoma from TCGA, separated based on BRAF and NRAS mutation status. Data were analyzed with a two-way ANOVA and no significant differences were found.

Figure S2. Diagram showing strategy for development of *Tyr-Cre^{ER}::Braf^{V600E}::Pten$^{-/-}$::mT/mG$^{fl/fl}$::Cxcr2$^{fl/fl}$* and *Tyr-Cre^{ER+}::NRas^{Q61R}::Ink4a$^{-/-}$::Cxcr2$^{fl/fl}$* mice (A, B, figures created using Biorender). Image of GFP-expressing *Braf^{V600E}/Pten$^{-/-}$* tumors *in vivo* and via histology (C). Immediately after 4HT administration, single cells were isolated from the mouse skin and stained with anti-CXCR2 for FACS analysis of CD45- GFP+ cells. The loss of CXCR2 is apparent in the *Cxcr2*$^{fl/fl}$ skin (D). FACS analysis of GFP+ cells after tumors have formed indicates that ~30% of GFP+ cells in the *Cxcr2*$^{fl/fl}$ tumors are positive for CXCR2 staining, compared to ~65% in the *Cxcr2^{WT}* tumors (E). Immunohistochemistry *Cxcr2^{WT}* and *Cxcr2$^{-/-}$* tumors shows approximately equivalent staining of the melanocyte/melanoma markers SOX10 and S100 in both tumors. We also see that membranous CXCR2 is higher in the *Cxcr2^{WT}* tumors.

Figure S3. Heat maps of the most differentially expressed genes in tumors from the *Braf^{V600E}/Pten$^{-/-}$* mice with or without loss of CXCR2 in tumor cells (A), with control or SX-682 treatment (B), and in MelanA, B16F0, and B16F10 cells treated with DMSO or SX-682 (C). Tumor suppressive genes are listed in red, genes involved in growth are in green, immune related genes are in blue, differentiation/stemness genes are in purple, and motility and cell adhesion genes are in brown.

Figure S4. Heat map showing genes involved in growth that are suppressed (A) and genes involved in growth suppression that are induced (B) in *Braf*^{V600E}/*Pten*$^{-/-}$/*Cxcr2*$^{-/-}$ melanoma tumors. Red arrows indicate genes involved in growth/oncogenes (A) and inhibition of growth/tumor suppression (B).

Figure S5. Additional data from analysis of immune cells and cytokines expressed by *Braf/Pten*$^{-}$/*Cxcr2*$^{-/-}$ and *Braf/Pten/Cxcr2*^{WT} tumors. mMCPCounter predicted infiltrate of B-derived cells, memory B cells, neutrophils, endothelial cells, mast cells, basophils, eosinophils, and blood vessels (A). FACS analysis of peripheral blood CD45+ cells from *Braf/Pten/Cxcr2*$^{-/-}$ and *Braf/Pten/Cxcr2*^{WT} mice (B). Results of cytokine array of tumor lysates from *Braf/Pten/Cxcr2*$^{-/-}$ and *Braf/Pten/Cxcr2*^{WT} melanoma tumors, expressed as fold change in *Cxcr2*$^{-/-}$ compared to *Cxcr2*^{WT} (CCL20 removed and shown in Figure 3D to allow proper visualization) (C). FACS analysis of CD45+ cells from *Braf/Pten/Cxcr2*$^{-/-}$ and *Braf/Pten/Cxcr2*^{WT} tumors (D). Statistical analyses: (A), (C), (D) Welch's t-test

Figure S6. Immunohistochemical staining was performed for Iba (a macrophage marker), CD3+ (total) T cells, CD4+ T cells, and CD8+T cells. As seen in FACS analysis, IHC indicates that there are approximately equal amounts of Iba+ macrophages and CD4+ T cells between the genotypes, with an increase in CD8+ cells in *Braf/Pten/Cxcr2*$^{-/-}$ tumors (A). Peripheral blood from *Braf/Pten/Cxcr2*$^{-/-}$ and *Braf/Pten/Cxcr2*^{WT} mice were analyzed via FACS and revealed no baseline differences in immune cell populations (B). Statistical analysis: (B) Welch's t-test

Figure S7. Additional data from analysis of immune cells from SX-682-treated *Braf*^{V600E}/*Pten*$^{-/-}$/*Cxcr2*^{WT} tumors. mMCPCounter predictions for T cells, B-derived cells, memory B cells, NK cells, endothelial, monocytes, macrophages, fibroblasts, lymphatics,

mast, basophils, eosinophils, and blood vessels (A). FACS analysis of peripheral blood CD45+ cells from tumor-bearing mice fed SX-682 or control chow (B). FACS analysis of CD45+ cells in SX-682 treated or control tumors (C).

To determine the hematological effects of SX-682, toxicology studies were performed at the IIT Research Institute (Chicago, IL). This study was conducted in compliance with the U.S. Food and Drug Administration (FDA) Good Laboratory Practice (GLP) Regulations (Code of Federal Regulations Title 21 Part 58). SX-682 was administered orally for 28 consecutive days (followed by 14 days of recovery) to male and female CD® IGS rats. The study consisted of four groups: Group 1, vehicle control (19 rats/sex); Group 2, 50 mg/kg/day SX-682 (17 rats/sex); Group 3, 150 mg/kg/day SX-682 (17 rats/sex); and Group 4, 250 mg/kg/day SX-682 (22 rats/sex). Ten rats/sex/group were necropsied on Study Day 29 (terminal necropsy) and 5 rats/sex in Groups 1 and 4 were necropsied on Study Day 43 (recovery necropsy). Rats subjected to plasma drug level and toxicokinetic analyses (3 rats/sex in Group 1 and 6 rats/sex in Groups 2-4) were bled at approximately 0.5, 1, 2, 4, 8, and 24 hours after the 1st and 28th dose. Consistent with the expected mechanism of SX-682, there was a dose-dependent but reversible reduction in absolute neutrophil count of no toxicological significance (D).C56BL/6 mice were treated with 50mg/kg SX-682 daily via oral gavage for 4 days prior to analysis of peripheral blood leukocytes via FACS. SX-682 reduced the percentage of Ly6G+ cells that were CD14+ (p=0.04) and increased the percentage of CD45+ Cells that were CD19+ (p=0.026) (E).

Statistical analyses: (A), (B), (C), (D) Welch's t-test

Figure S8. Effects of SX-682 on MelanA, B16F0, and B16F10 cell lines. MelanA, B16F0, and B16F10 cells were treated with SX-682 (5µM) or DMSO, and cell number was

evaluated at days 1-4 post treatment. Data were analyzed on a natural log scale and compared using two-way ANOVA with BH correction for multiple tests (A). Cell lines were treated for 24 hours with SX-682 or DMSO prior to RNA isolation and sequencing. A heat map shows that genes were commonly suppressed or induced in all three cell lines (B). Reverse-phase phosphoproteome analysis (RPPA) was also performed on cells treated for 24 hours with SX-682 or DMSO. Volcano plot showing phosphoproteins commonly altered by SX-682 treatment in MelanA, B16F0, and B16F10 cells (C). A heat map showing differential expression of commonly suppressed or induced phosphoproteins in response to treatment with 5μM SX-682 for 24 hours (D). Cytokine array of cell lysates from MelanA, B16F0, and B16F10 cells treated with SX-682 (5μM) for 24 hours as compared to those treated with DMSO (E).

Figure S9. Expression of *Tfcp2l1*-related genes and tumor markers based on RNAseq analysis in $Braf^{V600E}/Pten^{-/-}/Cxcr2^{-/-}$ tumors, $Braf^{V600E}/Pten^{-/-}$ tumors treated with SX-682, compared to appropriate controls. Statistical analysis: Welch's t-test

Figure S10. Expression of Tfcp2l1-related genes based upon RT-PCR analysis. MelanA, B16F0, and B16F10 cells were treated with SX-682 (5μM) for 24 hours prior to RNA extraction, quantification, and RT-PCR using primers specific for *Tfcp2L1, Foxd3, Sox2, Sox10, Notch1, Hmga2, Mitf, Klf4, Myc, Nanog, Esrrb and Tyr*. Data are plotted as fold-change compared to DMSO treated cultures. Statistical analysis: Welch's t-test

Figure S11. WGCNA orders genes into a dendrogram by their co-expression profiles across the samples. Gene modules at the bottom of the dendrogram show assignment into distinct clusters of co-expressed genes (A). Module eigengene expression value shown as a heatmap across WT and KO samples (B). Feature selection of modules by

ANOVA F-value (FDR-adjusted p-value). Six of 10 WGCNA gene modules can distinguish between conditions (C). Eigengene expression value across WT and KO samples shown as a bar-plot and significance of transcription factors (ordered by FDR-adjusted p-value) within each module for the six significant gene modules from panel D: blue, turquoise, brown, green, yellow, and red. These TFs are best at distinguishing between KO and WT samples in each module (D).

Figure S12. ChIPseq analysis of changes in gene promoter binding of TFCP2L1 antibody in B16F0 cells in response to SX-682 treatment. B16F0 cells were cultured overnight with either DMSO or SX-682. Cell extracts were incubated with anti-IgG or anti-TFCP2L1, cross-linked with a reversible cross-linker, and prepared and prepared for ChIP-seq analysis. Metascape analysis was used to examine enriched terms across input gene lists of SX-682 minus DMSO, colored by p-values. Top terms associated with TFCP2L1 bound promoters where gene expression was increased (A) and decreased (B) were identified. Metascape Analysis was used to compare the data sets from RPPA, ChIP, and RNAseq analyses of B16F0 cells treated with SX-682 as compared to respective controls (C). Cluster analysis of genes identified as regulated by SX-682 by ChIPseq, RNAseq, or RPPA, with commonly enriched GO terms across all three methods (D). Genes in Cluster analysis of S11D were colored by MCODE ID (E).

Figure S13. NHEM cells were cultured in melanocyte growth medium containing vehicle or CXCL1 (100ng/ml), or CXCL1 (100ng/ml) with 5 μM SX-682. After 5 days of culture, the cell number was determined, and total RNA was extracted for RNAseq analysis. Effects of SX-682 on cell growth in vitro (A). Heatmap for RNAseq on NHEM. Five days after NHEMs were cultured with SX-682 and/or CXCL1, RNA was extracted and

subjected to RNAseq analysis(B). Expression values for key genes associated with response to CXCL1 or CXCL1 and SX-682 (C).

Table S1. Comparison of Cytokine, Chemokine, and Interleukin Expression Following CXCL1 or CXCL1+SX-682 Treatment. Key cytokines induced are highlighted in red and reduced are highlighted in green.

Table S2. Comparison of TNF-Related Cytokines and Interferon Expression Following CXCL1 or CXCL1+SX-682 Treatment. Key cytokines induced are highlighted in red and reduced are highlighted in green.

Table S3. Preclinical validations. *Activity P<0.05. **time after tumor initiation. Abbreviations: CPPSML, PB-Cre+ PtenL/L p53L/L Smad4L/L mTmGL/+ LSL-LUCL/+, develops age-dependent green fluorescent protein (GFP+)LUC+ prostate cancer; ET, engineered T cells; G, genetically engineered; ICB1, immune checkpoint blockade (ICB) with anti-PD1 and anti-CTLA-4 cocktail; ICB2, anti-LAG3 and agonist-41BB ; KIL, murine NK cell line; MC38K and MC38-CEA, syngeneic MC38 colorectal cancer cell line with enforced expression of KRASG12D and CEA, respectively, NT, not tested; S, syngeneic; X, xenograft.

Supplemental References:

1. Lu X, Horner JW, Paul E, Shang X, Troncoso P, Deng P, et al. Effective combinatorial immunotherapy for castration-resistant prostate cancer. Nature. 2017;543(7647):728-32.
2. Yang J, Yan C, Vilgelm AE, Chen SC, Ayers GD, Johnson CA, et al. Targeted Deletion of CXCR2 in Myeloid Cells Alters the Tumor Immune Environment to Improve Antitumor Immunity. Cancer Immunol Res. 2021;9(2):200-13.
3. Kargl J, Zhu X, Zhang H, Yang GHY, Friesen TJ, Shipley M, et al. Neutrophil content predicts lymphocyte depletion and anti-PD1 treatment failure in NSCLC. JCI Insight. 2019;4(24).
4. Sun L, Clavijo PE, Robbins Y, Patel P, Friedman J, Greene S, et al. Inhibiting myeloid-derived suppressor cell trafficking enhances T cell immunotherapy. JCI Insight. 2019;4(7).
5. Liao W, Overman MJ, Boutin AT, Shang X, Zhao D, Dey P, et al. KRAS-IRF2 Axis Drives Immune Suppression and Immune Therapy Resistance in Colorectal Cancer. Cancer Cell. 2019;35(4):559-72 e7.
6. Greene S, Robbins Y, Mydlarz WK, Huynh AP, Schmitt NC, Friedman J, et al. Inhibition of MDSC Trafficking with SX-682, a CXCR1/2 Inhibitor, Enhances NK-Cell Immunotherapy in Head and Neck Cancer Models. Clin Cancer Res. 2020;26(6):1420-31.
7. Horn LA, Riskin J, Hempel HA, Fousek K, Lind H, Hamilton DH, et al. Simultaneous inhibition of CXCR1/2, TGF-beta, and PD-L1 remodels the tumor and its microenvironment to drive antitumor immunity. J Immunother Cancer. 2020;8(1).
8. Horn LA, Fousek K, Hamilton DH, Hodge JW, Zebala JA, Maeda DY, et al. Vaccine Increases the Diversity and Activation of Intratumoral T Cells in the Context of Combination Immunotherapy. Cancers (Basel). 2021;13(5).
9. Sinha VC, Rinkenbaugh AL, Xu M, Zhou X, Zhang X, Jeter-Jones S, et al. Single-cell evaluation reveals shifts in the tumor-immune niches that shape and maintain aggressive lesions in the breast. Nat Commun. 2021;12(1):5024.
10. Tang KH, Li S, Khodadadi-Jamayran A, Jen J, Han H, Guidry K, et al. Combined Inhibition of SHP2 and CXCR1/2 Promotes Antitumor T-cell Response in NSCLC. Cancer Discov. 2022;12(1):47-61.
11. Gulhati P, Schalck A, Jiang S, Shang X, Wu CJ, Hou P, et al. Targeting T cell checkpoints 41BB and LAG3 and myeloid cell CXCR1/CXCR2 results in antitumor immunity and durable response in pancreatic cancer. Nat Cancer. 2023;4(1):62-80.

Supplementary Methods:

Analysis of patient sequencing data.

RNA-Seq analysis of CXCL1, 2, 3, 5, and 8 from utilized patient nevi and melanoma samples available at NCBI Gene Expression Omnibus (GEO) under accession number GSE112509, including human nevi (n=23) and melanoma (n=57) samples. Bulk RNA-Seq analysis was performed as described previously (1). Differential mRNA gene expression was determined and normalized with the DESeq2 tool (Love et al., 2014) (Figure 1A, 1B). To analyze patient overall survival in reference to high or low CXCR2 expression, the Cancer Genome Atlas (TCGA) skin cutaneous melanoma (SKCM) dataset was analyzed using the Gene Expression Profiling Interactive Analysis (GEPIA) program. To generate the overall survival plot, the upper and lower quartiles of *CXCR2* expression were used to stratify the groups (Figure 1C). For analysis of the CXCR2 expression level predictive value in therapeutic response to immune checkpoint blockade in melanoma patients a Kaplan-Meier plot of overall survival was constructed by Tumor Immune Dysfunction and Exclusion (TIDE, http://tide.dfci.harvard.edu/) platform in patients with melanoma (2) stratified by CXCR2 z-scores of RNA-Seq normalized count. The P value was calculated by testing the association between prediction scores and overall survival with the two-sided Wald test in a Cox-PH regression (3) (Figure 1D, E).

Cell lines `

B16F0 (Catalog number: CRL-6322) and B16F10 (Catalog number: CCL-6475, ATCC) are spontaneous murine melanoma cell lines derived from a C57BL/6J mouse**.** They are tumorigenic and metastatic clones, respectively. Melan-A is a nontumorigenic murine cell

line that is syngeneic with B16 melanoma lines (Bennett et al., 1987). Normal human epidermal melanocytes (NHEMs) were purchased from Lonza (#CC-2504) and cultured in melanocyte growth medium including the MM-4 Bullet kit (Lonza, #CC-2349). To examine the regulation of CXCL1/CXCR2 signaling in NHEM, these cells were stimulated with either vehicle, CXCL1 (100ng/ml) or CXCL1+SX692 (5µM) for 24 hours. MelanA and B16 cells were cultured in DMEM/F12 medium (Gibco, #11330-032) containing 10% FBS (Sigma, PAA, A11-201). After 5 days of culture, the culture medium was removed, cells were trypsinized, aspirated, and collected by centrifugation, resuspended in serum containing medium, and the cell number was determined by Countess II (Invitrogen, C10228). Simultaneously, aliquots of cells were permeabilized and stained with Pacific Blue-KI67 and analyzed by flow cytometry. Experiments were conducted in duplicate and repeated once. Data were statistically analyzed with the Student's t-test. In other experiments, NHEMs were treated with CXCL1 (100ng/ml) or vehicle, or with SX-682 and CXCL1 for 24 hours before RNA was extracted and processed for RNAseq analysis. All cell lines are tested monthly for mycoplasma using a PCR detection system.

Mouse studies with the CXCR1/CXCR2 Inhibitor, SX-682

To evaluate the effect of the dual CXCR1/CXCR2 inhibitor SX-682 on tumor growth, $Braf^{V600E}/PTEN^{-/-}$ mice were fed with either SX-682 chow (0.756 g/kg of SX-682, Syntrix Pharmaceuticals, Inc) or control chow for two weeks prior to tumor induction via topical administration of 2µl of 5mM 4-HT onto the dorsal skin of 4-week-old mice for three successive days. The mice continued to be fed on the SX-682 chow or control chow until tumors grew to a volume of 1.5 cm^3 or required euthanasia due to ulceration or another

humane endpoint. *NRas*^{Q61R}/*Ink4a*$^{-/-}$ mice pups exposed to 4-HT and UV irradiation were weaned at 21 days and placed on SX-682 or control chow. Tumor volume was measured by microcalipers and tumor number for each mouse was counted after a period of 5 months.

To define the impacts of SX-682 on the peripheral immune populations of non-tumor bearing mice, 5 wildtype female C57BL/6 mice were treated daily with 50mg/kg SX-682 via oral gavage for 4 days prior to FACS analysis of peripheral immune populations as described in the flow cytometry methods.

Evaluation of SX-682 Toxicity

To determine the hematological effects of SX-682, toxicology studies were performed at the IIT Research Institute (Chicago, IL). This study was conducted in compliance with the U.S. Food and Drug Administration (FDA) Good Laboratory Practice (GLP) Regulations (Code of Federal Regulations Title 21 Part 58). SX-682 was administered orally for 28 consecutive days (followed by 14 days of recovery) to male and female CD® IGS rats. The study consisted of four groups: Group 1, vehicle control (19 rats/sex); Group 2, 50 mg/kg/day SX-682 (17 rats/sex); Group 3, 150 mg/kg/day SX-682 (17 rats/sex); and Group 4, 250 mg/kg/day SX-682 (22 rats/sex). Ten rats/sex/group were necropsied on Study Day 29 (terminal necropsy) and 5 rats/sex in Groups 1 and 4 were necropsied on Study Day 43 (recovery necropsy). Rats subjected to plasma drug level and toxicokinetic analyses (3 rats/sex in Group 1 and 6 rats/sex in Groups 2-4) were bled at approximately 0.5, 1, 2, 4, 8, and 24 hours after the 1st and 28th dose.

Flow cytometry analysis and antibodies

For flow cytometry analyses, tissues were minced on a programmable dissociator and digested with an enzyme solution of collagenase 1 (1,500 CDU, CAT#234153, Calbiochem), dispase II (1 mg/mL, CAT#13689500, Roche), and DNase 1 (0.1 mg/mL, CAT#260913, Calbiochem). The details of staining and flow cytometry analyses protocols can be found in our previous published methodology (4). Mouse tumors were cut into 1- to 2-mm slices and digested in buffer containing 2 mg/mL collagenase and 0.1 mg/mL DNase I. Digested tumors were passed through a 70-mm strainer to obtain a single-cell suspension. Mouse spleens were pressed through 40 mm strainer using syringe plunger to obtain a single-cell suspension. Red blood cells present in whole blood or cell preparations were removed using ACK Lysing Buffer (CAT#RGF-3015, KD Medical) prior to the staining. Cells were incubated with Ghost Dye TM Violet 510 (Tonbo Biosciences), an amine reactive viability dye used to discriminate live/dead cells and washed with FACS buffer (PBS containing 2% v/v FBS). After blocking Fc receptors with anti-mouse CD16/CD32 mAb in FACS buffer for 15 minutes, cells were incubated with mAbs (BioLegend) to mouse CD45-APC/Cy7, CD3-Alexa Fluor 594, CD4-FITC, CD8a-PE, CD44-APC, CD62L-Alexa Fluor 700, CD25-Percp/Cy5.5, CD69-pacific blue; CD45-Alexa Fluor 488, CD11b-PE, F4/80-Brilliant violet 421, Ly6C-APC, Ly6G-PE/Cy7, MHC II-Alexa Fluor 700, CD206-Percep/Cy5.5, etc. 1 hour on ice.

For flow cytometric analysis of CXCR2 expression in melanocytes following 4HT application or tumor formation, single cells were isolated from skins or tumors of mice and stained with anti-CXCR2 Ab (MAB2164-sp, R&D Systems) at 1:1000 for 2h, followed by

secondary antibody application of Alexa Fluor 647-conjugated rabbit anti-rat IgG (#312-605-003, Jackson ImmunoResearch Lab Inc) at 1:10,000 for 30 min.

Cells were washed twice in FACS buffer and data acquired with FACSCanto II (Becton Dickenson). For intracellular staining after surface staining, cells were fixing/permeabilizing using Transcription Factor Buffer Set (Cat:562674, BD Pharmingen) per manufacture's protocol. FACS data were analyzed using FlowJo software (Version 10.1).

Immunohistochemistry

All slides were placed on the Leica Bond-Rx IHC stainer. All steps besides dehydration, clearing and coverslipping are performed on the Bond-Rx. Slides are deparaffinized prior to stain specific protocols:

CD3: Heat induced antigen retrieval was performed on the Bond Max using their Epitope Retrieval 2 solution for 5 minutes. Slides were incubated with anti-CD3 (Cat. No# 019-19741, FujiFilm, Madison, WI) for one hour at a 1:1000 dilution. The Bond Polymer Refine Red Detection system (cat# DS9390, Leica Biosystems, Deerpark, IL) and DAKO/Agilent Rabbit-HRP EnVision+ system was used for visualization.

CD4: Heat induced antigen retrieval was performed on the Bond Max using their Epitope Retrieval 2 solution for 20 minutes. Slides were incubated with anti-CD4 (Cat. No.: HS-360 117, Synaptic Systems GmbH, Goettingen, Germany) for one hour at a 1:1500 dilution and then incubated in a rabbit anti-rat secondary (BA-4001, Vector Laboratories, Inc.) for 15mins at a 1:2000 dilution. The Bond Polymer Refine Red Detection system (cat# DS9390, Leica Biosystems, Deerpark, IL) was used for visualization.

CD8: Heat induced antigen retrieval was performed on the Bond Max using their Epitope Retrieval 2 solution for 20 minutes. Slides were incubated with anti-CD8 (Cat HS-361 003, Synaptic Systems GmbH, Goettingen, Germany) for one hour at a 1:1000 dilution. The Bond Polymer Refine Red Detection system (cat# DS9390, Leica Biosystems, Deerpark, IL) was used for visualization.

CXCR2: Heat induced antigen retrieval was performed on the Bond Max using their Epitope Retrieval 2 solution for 20 minutes. Slides were incubated with anti-CXCR2/IL-8 (Catalog #MAB2164, R&D Systems, Minneapolis, MN) for one hour at a 1:200 dilution and followed by a biotinylated anti-rat (Cat.# AI-4001-.5, Vector Laboratories, Inc., Burlingame, CA) for 15 minutes at a 1:2000 dilution. The Bond Polymer Refine detection system was used for visualization.

S100: . Heat induced antigen retrieval was performed on the Bond Max using their Epitope Retrieval 2 solution for 20 minutes. Slides were placed in a Protein Block (Ref# x0909, DAKO, Carpinteria, CA) for 10 minutes. The sections were incubated with anti-S100 (GA50461-2, Agilent(DAKO), Santa Clara, CA) at a dilution of 1:6000 for 60 minutes. The Bond Polymer Refine Red Detection system (cat# DS9390, Leica Biosystems, Deerpark, IL) was used for visualization.

SOX10: Heat induced antigen retrieval was performed on the Bond Max using their Epitope Retrieval 2 solution for 10 minutes. Slides were incubated with anti-SOX10 (Catalog #104225-1-AP, Proteintech Group, Inc., Rosemont, IL) for one hour at a 1:1250 dilution. The Bond Polymer Refine Red Detection system (cat#DS9390, Leica Biosystems, Newcastle Upon Tyne, United Kingdom) was used for visualization.

CXCR2-SOX10 Co-Staining: Heat induced antigen retrieval was performed on the Bond Max using their Epitope Retrieval 2 solution for 20 minutes. Slides were incubated with anti-CXCR2/IL-8 (Catalog #MAB2164, R&D Systems, Minneapolis, MN) for one hour at a 1:200 dilution and followed by a biotinylated anti-rat (Cat.# AI-4001-.5, Vector Laboratories, Inc., Burlingame, CA) for 15 minutes at a 1:2000 dilution. The Bond Refine (DS9800, Buffalo Grove, IL, USA) detection system was used for visualization. The sections were then incubated with anti-SOX10 (Catalog #104225-1-AP, Proteintech Group, Inc., Rosemont, IL) for one hour at a 1:1250 dilution. The Bond Polymer Refine Red Detection system (cat#DS9390, Leica Biosystems, Newcastle Upon Tyne, United Kingdom) was used for visualization.

Iba1: Heat induced antigen retrieval was performed on the Bond Max using their Epitope Retrieval 2 solution for 5 minutes. Slides were incubated with anti-Iba1 (Cat. No# 019-19741, FujiFilm, Madison, WI) for one hour at a 1:1000 dilution. The Bond Polymer Refine Red Detection system (cat# DS9390, Leica Biosystems, Deerpark, IL) used for visualization.

All slides were then dehydrated, cleared, and coverslipped.

Cytokine Array

Tumor lysate or supernatant of cultured cells were prepared as previously described (Yang et al., 2021). For cultured cell lines, $3x10^5$ cells were plated per well into 6-well plates and incubated overnight. Cells were then treated for 24 hours with 5μM SX-682 (Syntrix) or DMSO prior to supernatant collection. Supernatants were centrifuged to remove cell debris prior to analysis, and both tumor lysates and cell supernatant were subjected to analysis with the Raybio Mouse Cytokine Antibody Array G-Series 3 (Cat#

AAM-CYT-G3-8, RayBiotech) per manufacturer's protocol. The glass chip was scanned on the Cy3 channel of a GenoPix 4000B scanner (Genopix 6.1, Molecular Devices, Sunnyvale, CA). For each spot, the net density was determined by subtracting the background. The relative fold difference in cytokine amount was determined in reference to the amount present on the control samples.

RNA extraction and RNAseq analysis

RNA was extracted from tumor tissues or cultured cells using the RNeasy Plus Mini Kit (Cat#74134, Qiagen) per manufacturer's protocol. RNAseq was performed using an Illumina next-generation sequencing platform at the Vanderbilt University Medical Center VANTAGE (Vanderbilt Technologies for Advanced Genomics) Core Facility. Adapters were trimmed by Cutadapt. After trimming, reads were mapped to the mouse genome GRCm38.p6 using STAR and quantified by featureCounts. DESeq2 was used to normalize expression prior to analysis unless otherwise stated. Data were analyzed using GSEA (4.1.0). Gene sets analyzed include: GOBP_CD4_POSITIVE_ALPHA_ BETA_T_CELL_ACTIVATION, GOBP_LYMPHOCYTE_ACTIVATION, GOBP_ LEUKOCYTE_PROLIFERATION, GOBP_LYMPHOCYTE_ANERGY, GOBP_IMMUNE_ RESPONSE, GOBP_STEM_CELL_DIFFERENTIATION, GOBP_ADAPTIVE_IMMUNE_ RESPONSE, GOBP_STEM_CELL_DIVISION, GOBP_CD8_POSITIVE_ALPHA_BETA_ T_CELL_ACTIVATION, GOBP_T_CELL_MEDIATED_IMMUNE_RESPONSE_TO_TUMOR_CELL, GOBP_IMMUNE_RESPONSE_TO_TUMOR_CELL, GOBP_ANTIGEN_ PROCESSING_ AND_ PRESENTATION, GOBP_CD8_POSITIVE_ALPHA_BETA_T_ CELL_PROLIFERATION, GOBP_T_CELL_MEDIATED_CYTOTOXICITY, GOBP_ CELL_CYCLE_PROCESS, GOBP_CELL_CYCLE, GOBP_CELL_CYCLE_G1_S_ PHASE_TRANSITION, and GOBP_MELANOCYTE_PROLIFERATION (Human Molecular Signatures Database-MSigDB).

For GSEA, 1000 permutations were performed and human orthologs were annotated with Mouse_Gene_Symbol_Remapping_Human_ Orthologs_MSigDB.v2022.1.Hs.chip. Phenotype permutation was used except when the sample size per phenotype was less than 7. In this case, we follow the GSEA recommendation to use gene set permutation.

For expression plots, DESeq2 normalized counts were transformed by adding one to the value (to retain 0s) and then taking the natural log to account for variance. Comparisons were made using a Mann-Whitney test where appropriate.

For murine microenvironment cell population counter (mMCPcounter) analysis, each count file of protein coding genes was normalized to transcripts per million (TPM) as recommended by the program. TPM was calculated by determining rate as the raw counts divided by the gene length, and then dividing each rate by the sum of all rates multiplied by 1e6. Following TPM normalization, data were transformed utilizing log2(TPM normalized expression +1). The mMCPcounter (v1.1.0) package was installed and the function mMCPcounter.estimate was used to determine predicted immune cell infiltrate.

Weighted gene co-expression network analysis (WGCNA)

WGCNA (5) was performed on log-transformed, normalized RNA-seq data of 16382 genes from 15 mouse tumor samples (n = 7 WT mice and n = 8 KO mice) using the R package “WGCNA.” A signed network was generated, such that only positively correlated genes were grouped into modules (negative correlations are given a score of 0), as follows. The function pickSoftThreshold was used to determine the exponent of the correlation coefficients matrix which, when used as weights of network connections, best produces a scale-free network. A power value of 20 was chosen. A topological overlap matrix of network adjacencies between genes was then generated with the adjacency

and TOMsimilarity functions, using the Pearson correlation function, which gave a distance measure to be used with average-linkage hierarchical clustering. The WGCNA function cutTreeDynamic was used, with minimum module size of 100, deepSplit = 2, and pamRespectsDendro = FALSE, to generate modules of co-expressed genes. Differentially expressed gene modules across WT and KO conditions were determined using an ANOVA statistical test with FDR correction using the python function selectFDR from the Sci-Kit Learn package. Six gene modules could significantly distinguish between the WT and KO tumors (FDR-adjusted p-value < 0.05) (6).

Identification of Transcription Factor Network Structure

To determine the most important transcription factors (TFs) that both distinguish WT and KO conditions and were central to a gene module, we calculated the module membership value, or kME, for each TF, a measure of correlation of its gene expression with the module eigengene from WGCNA. TFs were filtered to those with a significant FDR-adjusted p-value for the ANOVA between WT and KO (< 0.05). We then chose the top 40 significant TFs by kME for each of the six significant gene modules to find the most central TFs for each module.

Epigenome mapping by ChIPmentation for TCPF21

ChIPmentation was performed as previously described (7,8) with some modifications. Cells were treated with 5μM SX-682 or DMSO vehicle for 24 hours, collected by trypsinization, and resuspended in PBS. Next, cells were fixed with fresh formaldehyde at final concentration of 1% for 10 min at room temperature. Glycine was added to final

concentration of 0.125 M to quench the reaction. Cells were resuspended at $1x10^7$/ml in sonication buffer (10 mM Tris-HCl pH 8.0, 2 mM EDTA pH 8.0, 1% SDS, with Protease Inhibitor Cocktail (Roche) and PhosSTOP™ Phosphatase Inhibitor Cocktail (Roche) and sonicated for 25-30 sec with Diagenode One sonicator in a 50 ul Bioruptor® One Microfluidic Chip till most DNA fragments were in the range of 200 to 700 bp. After sonication, the lysate was adjusted to RIPA buffer conditions (10 mM Tris-HCl pH 8.0, 1 mM EDTA pH 8.0, 140 mM NaCl, 1% Triton X-100, 0.1% SDS, 0.1% sodium deoxycholate, with Protease Inhibitor Cocktail and PhosSTOP™ Phosphatase Inhibitor Cocktail). For each immunoprecipitation, lysate from $2x10^6$ cells and 6 µg of anti-TFCP2L1 antibody (Boster Bio) were used. 6.0 µg of normal rabbit IgG was used as a control. 40µl Dynabeads ™ Protein A (Invitrogen) were used in each immunoprecipitation. After immunoprecipitation, beads were washed twice with RIPA low-salt buffer, twice with RIPA high-salt buffer, twice RIPA lithium-chloride buffer, and once with 10 mM Tris-HCl buffer (pH 8.0). Illumina sequencing adapters were added on bead-bound DNA fragments via tagmetation by using Illumina Tagment DNA TDE1 Enzyme and Buffer Kits (Illumina). After tagmentation, bead-bound DNA fragments were extracted by reversing the crosslink and proteinase K digestion. DNA fragments were purified with AMPure XP beads (Beckman Coulter). qPCR was performed first with KAPA HiFi HotStart Ready Mix and a pair of Nextera custom primers to determine the optimum number of PCR cycles for the DNA library preparation for each immunoprecipitation. The final enriched DNA library for each immunoprecipitation was created by PCR with KAPA HiFi HotStart Ready Mix and Nextera custom primers using the optimum number of PCR cycles determined by qPCR. The enriched DNA library was purified with AMPure XP beads, and size-selection was

performed by controlling the concentration of ethanol in the AMPure XP beads and DNA mixture. Acquired DNA libraries were sequenced by the Illumina NovaSeq 6000 platform at Vanderbilt Technologies for Advanced Genomics core laboratory. ChIPmentation reads were aligned to the mouse reference genome mm10 using Bowtie2 (9). Peaks for each sample were called by MACS2 with an FDR cutoff of 0.01 and the corresponding IgG input as the control (10-12). Peaks were annotated using Homer (http://homer.ucsd.edu/homer/) and assigned to their closest genes. Enriched motifs were identified by the Homer command findMotifsGenome with the default region size and the motif length (-size 200 and -len 8, 10, 12). Genes that had TFCP2L1 bound to their 5' promoter in the DMSO and SX-682-treated samples were selected and analyzed further using Metascape software (13). Comparisons were made among RPPA, RNAseq, and ChIPseq data sets for B16F0 cells using Metascape software to identify commonly enriched pathways.

RT-qPCR

B16F0, B16F10, and MelanA cells were treated with 5µM SX-682 or DMSO for 12 hours prior to collection and RNA extraction with Trizol. cDNA was generated using a Reverse Transcription kit (Catalog number M510A, Promega), and qPCR was performed using a BioRad CFX-qPCR instrument and SsoAdvanced Universal SYBR Green Supermix (Cat: 172-5270, BioRad). Fold changes were calculated using the formula $2^{-(\Delta\Delta Ct)}$, where $\Delta\Delta Ct = \Delta Ct^{(SX\text{-}682)} - \Delta Ct^{(DMSO)}$. The Ct is the cycle at which the threshold line is crossed. Primers for β-actin, sense-ACATGGCATCATCACCAACTG and antisense-AGAATCCAACACGATGCCGG. TFCP2L1, sense-ACACTACAACCAGCACAACTC and

antisense-TGGTACTCTGTG TACTGCAGC. Sox10, sense-ACCTATCAGAGGTGGAGCTGAG and antisense-TGC TGTTCCTTCTTGACCTTG. MITF, sense-AGAGCAGCAGTTCTGCAGAGC and antisense-ATGGCTGGTGTCTGACTCACG. Nanog, sense-TCTGCTACTGAGATG CTCTGC and antisense-ACAGTCCGCATCTTCTGCTTC. FoxD3, sense-AGCGATA TGTCCGGCCAGACG and antisense-TGAGACTGGCCGTGATGAGCG. Notch1, sense-TGACTGTACTGAGAGCTCCTG and antisense-AAGTACCATAGCTGTCTTGGC. TLF4, sense-ATGGCTGTCAGCGACGCTCTG and antisense-TGTTACTGCTGCAAGCTG CAC. Slc4A1, sense-ACTGGAGAACATAATAGGACAG and antisense-AAGGTCAGG TAAGATAGATGTG. Esrrb, sense-AGTGCGAGTATATGCTTAATG and antisense-TGAATTGTCCTCTTGAAGAAG. Sox2, sense-ATGATGGAGACGGAGCTGAAG and antisense-TTGCTGATCTCCGAGTTGTGC. HMGA2, sense-TGCCACAGAAGCGAGGA CGCG and antisense-TCCTAGGTCTGCCTCTTGGCC.

Protein extraction and RPPA analysis

Melan-A, B16F0, and B16F10 cells were seeded at a density of $3x10^5$ per well in a 6-well plate overnight prior to 24-hour treatment with 5μM SX-682. Cells were then lysed in a buffer containing 1% Triton X-100, 50mM HEPES, pH 7.4, 150mM NaCl, 1mM EGTA, 100mM NaF, 1.5mM $MgCl_2$, 10mM Na pyrophosphate, 1mM Na_3VO_4, 10% glycerol, and freshly added protease inhibitors (Cat# 05056489001, Roche) and phosphatase inhibitors (Cat# 04906837001, Roche) in 2mL reinforced tubes (Cat# P000943-LYSKO-A, Precellys). The homogenization sets at 5000 and goes for 10 sec for cells and 30 sec for tissue in the Precellys 24 Tissue Homogenizer. Lysates were

centrifuged at 14,000rpm, 4°C for 2 minutes, and supernatants were collected. Protein concentration was adjusted to 1.5mg/mL and samples were stored in sample buffer (40% glycerol, 8% SDS, 0.25M Tris-HCL, pH 6.8. 10% β-mercaptoethanol is added just before use.) Samples were stored at -80°C until they were submitted to MD Anderson Cancer Center for processing and RPPA analysis of 480 proteins.

RPPA data were normalized and analyzed after a log2 transformation. All comparisons were performed using Welch's t-test. Changes in protein expression were considered significant based upon two criteria: p-value < 0.05 and a $\log_2$(fold change) of ±2 (equivalent to a fold change of ±4). Functional protein association networks are presented using STRING version 11.5.

Statistical analysis

Data were summarized in figures using either the mean ± standard deviation (SD) for error bar charts or median with the first and third quartiles for boxplots. The mean of two groups were compared using Welch's t-test. For a comparison of more than two group means, the one-way analysis of variance (ANOVA) with post-hoc Tukey's test was used. The mean difference between two groups by the other factor was assessed in the context of a two-way ANOVA. Pairwise differences between two groups were compared using model-based mean comparisons. The Benjamini and Hochberg (BH) was used to adjust p-value for multiple comparisons as noted in the text to control the within experiment false discovery rate to less than 5%. Data was analyzed on a natural log scale or rank-based scales to meet the normality assumptions of statistical tests as needed. Survival curves are estimated using the Kaplan-Meier method and compared between two groups using

the log-rank test. Levels of statistical significance are denoted * for p<0.05, ** for p<0.01, and *** for p<0.001, respectively. The analyses were performed using R version 4.1.2 or GraphPad Prism software. WGCNA statistical analysis was performed as described in the WGCNA methods.

References for Supplementary Methods:

1. Yan C and Richmond A. (2021). Hiding in the dark: pan-cancer characterization of expression and clinical relevance of CD40 to immune checkpoint blockade therapy. Mol Cancer 20: 146, 10.1186/s12943-021-01442-3

2. Fu J, Li K, Zhang W, Wan C, Zhang J, Jiang P, Liu XS. (2020). Large-scale public data reuse to model immunotherapy response and resistance. Genome Med 12:21 10.1186/s13073-020-0721-z.

3. Jiang P, Gu S, Pan D, Fu J, Sahu A, Hu X, Li Z, Traugh N, Bu X, Li B, et al: (2018). Signatures of T cell dysfunction and exclusion predict cancer immunotherapy response. Nat Med 24:1550-1558. 10.1038/s41591-018-0136-1.

4. Yang, J., Yan, C., Vilgelm, A.E., Chen, S.C., Ayers, G.D., Johnson, C.A., and Richmond, A. (2021). Targeted Deletion of CXCR2 in Myeloid Cells Alters the Tumor Immune Environment to Improve Antitumor Immunity. Cancer Immunol Res *9*, 200-213. 10.1158/2326-6066.CIR-20-0312.

5. Langfelder P and Horvath S (2008). WGCNA: an R package for weighted correlation network analysis. BMC Bioinformatics. 9:559. 10.1186/1471-2105-9-559

6. Wooten DJ, Groves SM, Tyson DR, Liu Q, Lim JS, Albert R, Lopez CF, Sage J, Quaranta V. 2019. Systems-level network modeling of Small Cell Lung Cancer subtypes identifies master regulators and destabilizers. Plos Comput Biol 15: e1007343. https://journals.plos.org/ploscompbiol/article?id=10.1371/journal.pcbi.1007343.

7. Xu W, Ye Y, Sharrocks AD, Zhang W, Chen X (2020). Genome-wide interrogation of protein-DNA interactions in mammalian cells using ChIPmentation. STAR Protoc. 1(3):100187 10.1016/j.xpro.2020.100187

8. Krausgruber T, Fortelny N, Fife-Gernedl V, Senekowitsch M, Schuster LC, Lercher A, Nemc A, Schmidl C, Rendeiro AF, Bergthaler A, and Bock C. (2020) Structural cells are key regulators of organ-specific immune responses. Nature. 583(7815):296-302. 10.1038/s41586-020-2424-4.

9. Langmead, B. and Salzberg, S.L. Fast gapped-read alignment with Bowtie 2. Nat Methods 2012;9(4):357-359. 10.1038/nmeth.1923.

10. Feng, J.Liu T Qin B, Zhang Y, Liu XS (2012). Identifying ChIP-seq enrichment using MACS. *Nature protocols*7(9):1728-1740. 10.1002/0471250953.bi0214s34.

11. Feng, J., Liu, T. and Zhang, Y. Using MACS to identify peaks from ChIP-Seq data. (2011). *Current protocols in bioinformatics.* Chapter 2:Unit 2 14. 10.1002/0471250953.bi0214s34.

12. Zhang, Y., Liu T, Meyer CA, Eeckhoute J, Johnson DS, Bernstein BE, Nusbaum C, Meyers RM, Brown M, Li W, Siu S. Model-based analysis of ChIP-Seq (MACS). *Genome biology* 2008;9(9):R137. 10.1186/gb-2008-9-9-r137

13. Zhou Y, Zhou B, Pache L, Chang M, Khodabakhshi A H, Tanaseichuk O, Benner C, Chanda SK. (2019). Metascape provides a biologist-oriented resource for the analysis of systems-level datasets. Nature Commun. 10: 1523-1563. 10.1038/s41467-019-09234-6.
